# World Allergy Organization (WAO) Diagnosis and Rationale for Action against Cow’s Milk Allergy (DRACMA) Guidelines update – IV – A quality appraisal with the AGREE II instrument

**DOI:** 10.1016/j.waojou.2021.100613

**Published:** 2022-03-02

**Authors:** Agata Stróżyk, Marek Ruszczyński, Andrea Horvath, Lamia Dahdah, Alessandro Fiocchi, Anna Nowak-Węgrzyn, Raanan Shamir, Jonathan Spergel, Yvan Vandenplas, Carina Venter, Hania Szajewska, the WAO DRACMA guideline group

**Affiliations:** a Department of Paediatrics, The Medical University of Warsaw, Warsaw, Poland; b Allergy Unit, Pediatric University Department, Bambino Gesù Children's Hospital, Rome, Italy; c Department of Pediatrics, NYU Grossman School of Medicine, Hassenfeld Childrens' Hospital, New York, NY, USA; d Institute for Gastroenterology, Nutrition and Liver Diseases, Schneider Children's Medical Center, Lea and Arieh Pickel Chair for Pediatric Research, Sackler Faculty of Medicine, Tel Aviv University, Petach Tikva, Israel; e Division of Allergy-Immunology, Children's Hospital of Philadelphia, Perelman School of Medicine at University of Pennsylvania, Philadelphia, PA, USA; f KidZ Health Castle, UZ Brussel, Vrije Universiteit Brussel, Brussels, Belgium; g Section of Allergy and Immunology, University of Colorado and Children's Hospital Colorado, Aurora, CO, USA; h Department of Pediatrics, Gastroenterology and Nutrition, Collegium Medicum, University of Warmia and Mazury, Olsztyn, Poland

**Keywords:** Children, Cow's milk allergy, Guidelines, AGREE II

## Abstract

**Background:**

Since the publication of The World Allergy Organization (WAO) Diagnosis and Rationale for Action against Cow's Milk Allergy (DRACMA) Guidelines in 2010, a number of other guidelines, expert opinions, and position papers relating to the management of cow's milk allergy (CMA) have been published. We aimed to systematically review the quality of the guidelines on CMA diagnosis and management in children and/or adults published between 2010 and 2020.

**Methods:**

The MEDLINE, EMBASE, ISI Web of Science, World Health Organization Global Index Medicus, and Turning Research into Practice databases as well as website guideline repositories were searched from January 2010 until May 2020. Any clinical practice recommendations and/or guidelines focusing on the diagnosis and management of CMA in children and/or adults developed or endorsed by professional scientific societies or organizations were included. The guidelines were evaluated using the Appraisal of Guidelines for Research and Evaluation (AGREE II) tool, a 23-item tool organized within 6 domains and 2 global rating items.

**Results:**

We included 12 guidelines; 8 were developed by national and 4 by international organizations. The quality scores for each domain varied: of all domains, the clarity of presentation domain had the highest median score (92%; Q1-Q3 81–100%), whereas rigor of development had the lowest median score (30%; Q1-Q3 15–67%). The median scores (Q1-Q3) for individual domains were as follows: scope and purpose 82% (70–99%), stakeholder involvement 63% (21–79%), rigor of development 30% (15–67%), clarity of presentation 92% (81–100%), applicability 68% (57–75%), and editorial independence 75% (69–100%). The median overall score was 70% (58–89%). Only 1 guideline (from the National Institute for Health and Care Excellence [NICE]) achieved top ratings (100%) in five domains and the overall score. Three guidelines (from the NICE, the British Society for Allergy & Clinical Immunology [BSACI] and WAO) achieved the highest ratings (100%) in at least 3 domains and the overall score.

**Conclusion:**

The majority of identified guidelines were of good or very good quality. However, the weakest point was the rigor of development domain, mostly due to unclear description of strengths and limitations of the body of evidence and the procedure for updating the guidelines.

## Introduction

Since the publication of the 2010 DRACMA guidelines, a number of other guidelines, expert opinions, and position papers for the management of CMA have been published. However, their quality has not been formally appraised. In 2016 a systematic review assessed the quality of guidelines on cow's milk allergy (CMA) published from 2010 through November 2015 using the Appraisal of Guidelines for Research and Evaluation (AGREE II) tool.^1^ Fifteen guidelines were included. Only the guidelines developed by recognized professional/scientific organizations such as the British Society for Allergy and Clinical Immunology (BSACI) and the European Academy of Allergy and Clinical Immunology (EAACI) were of the highest quality. In addition, the 2010 World Allergy Organization (WAO) Diagnosis and Rationale for Action against Cow's Milk Allergy (DRACMA) Guidelines,^2^ the only Grading of Recommendations Assessment, Development and Evaluation (GRADE) guidelines for CMA, were considered to be of high quality.

In 2018, the DRACMA panel committee re-assembled in order to update the DRACMA guidelines. The aim of this study was to systematically review the quality of the guidelines on CMA diagnosis and management in children and/or adults published from 2010 onwards, and to summarize specific recommendations.

**Figure:**
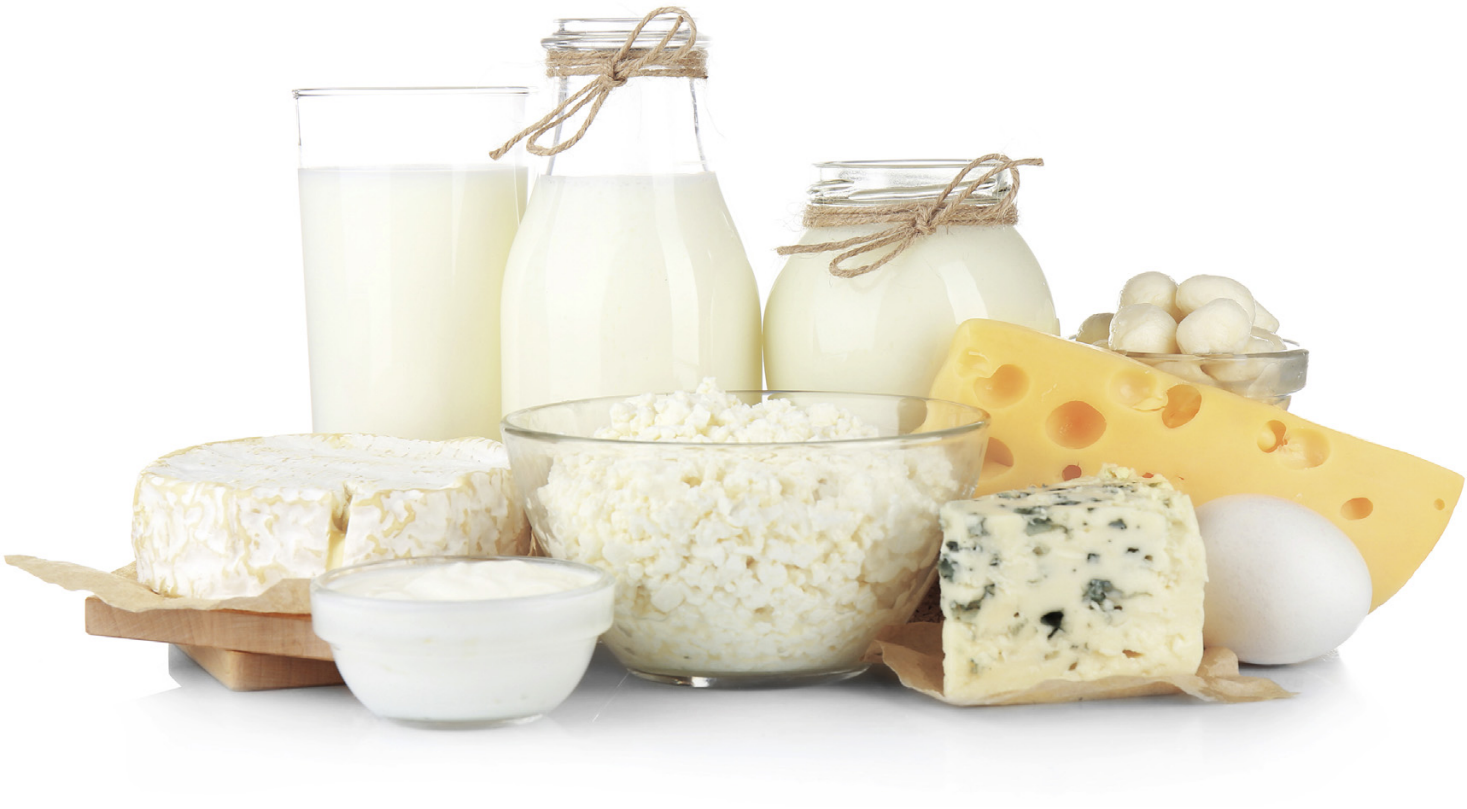

## Methods

The Preferred Reporting Items for Systematic Reviews and Meta-Analyses (PRISMA) statement^3^ was followed during each stage of this review. The protocol was pre-defined and submitted to PROSPERO; however, it was not accepted for registration, as it was assessed as being outside of the scope of included protocols due to the lack of at least 1 outcome of direct patient or clinical relevance. The AGREE II User's Manual^4^ was followed during the quality assessment of the included guidelines.

### Search for guidelines

The MEDLINE (through PubMed), EMBASE, ISI Web of Science (Thomson Web of Knowledge), World Health Organization Global Index Medicus (GIM) (https://www.globalindexmedicus.net/), and Turning Research into Practice (TRIP) (https://www.tripdatabase.com/) databases were searched from January 2010 up until May 2020, and then the search was updated in April 2021. The rationale for choosing 2010 as the start date was that this is the issue date of the DRACMA guidelines. However, we recognized that an update of any guidelines/recommendations is generally required from 2 to 5 years after the issue date,^5^ and therefore, some of the earlier guidelines could be outdated. MEDLINE and EMBASE were searched following a pre-specified search-strategy (see Supplemental Appendix 1). The websites of guideline repositories were also searched including: National Institute for Clinical Excellence (NICE, https://www.nice.org.uk/), The Guideline International Network (GIN, https://guidelines.ebmportal.com/), Scottish Intercollegiate Guidelines Network (SIGN) (https://www.sign.ac.uk), and Agency for Healthcare Research and Quality (AHRQ, https://www.ahrq.gov/).

References of all included guidelines and guideline publisher's websites were also searched for any supporting documents (ie, technical reports, methodological manuals).

The search was carried out independently by four reviewers (AS, AH, LD, and MR). No filters or restrictions other than English language were imposed.

### Eligibility criteria

#### Inclusion criteria & exclusion criteria

Any clinical practice recommendations and/or guidelines focusing on the diagnosis and management of CMA in children and/or adults developed or endorsed by recognized scientific societies or organizations were included. In case of an updated version of a guideline, only the most recent document was considered for inclusion. Guidelines were included, regardless of CMA mechanism (ie, IgE-mediated, non-IgE-mediated, mixed); however, if feasible, they were assessed separately. Guidelines focusing on food allergy or a single disease (eg, food protein-induced enterocolitis syndrome [FPIES]) were not considered for inclusion in this review, unless there was a section focusing explicitly on CMA or cow's milk proteins.

Consensus-based and expert opinion clinical practice guidelines, if not endorsed by recognized scientific or professional organizations, were excluded based on their limited generalizability as well as our limited capability to evaluate the level of expertise, that these publications represent, and the audience addressed. Guidelines focused on a single specific management option (eg, immunotherapy) or prevention were excluded. Guidelines which were ongoing or unpublished were also excluded.

### Data selection

As recommended, 4 reviewers (AS, AH, MR, and LD) screened the titles and abstracts of articles identified in the search to identify potentially eligible guidelines. The full texts of all potentially relevant articles were retrieved and critically assessed against the pre-defined inclusion criteria independently by each of the reviewers. Any discrepancies were first discussed by the 4 reviewers (AS, AH, MR, and HS).

Initially, members of the DRACMA panel not involved in the earlier process (AF, ANW, RS, JS, YV, CV, LD) provided their comments on the included and questionable documents and, if feasible, any unidentified papers, via an online survey using Google Forms. The list of excluded papers was also reviewed. Guidelines were included if at least 90% agreement was reached; in case of agreement ≤50%, a paper document was excluded. All of the comments were discussed. Then, all questionable documents (between 50% and 90% agreement) were put to a second vote by the members of DRACMA panel to determine eligibility for inclusion. Any discrepancies, as well as all other disagreements between the reviewers, were resolved through discussion until a consensus was reached.

### Data extraction

Three reviewers (AS, MR, and LD) independently extracted data from all included guidelines. The reviewers extracted the following information: title, year of publication, organization (country), level of guideline development (ie, local, regional, national, or international), financial support, and conflicts of interest (number of people who obtained financial support and/or had conflicts of interest/number of all authors). Data extraction was performed using data-extraction forms developed by the reviewers. Any discrepancies were discussed until a consensus was reached.

Specific recommendations were summarized in a comparative table, focusing on possible gaps and common messages. A “List of specific recommendations to be assessed” had been pre-specified in the protocol. If feasible, recommendations were extracted separately for IgE-mediated, non-IgE-mediated, and mixed CMA, as well for each age group (ie, children, adults).

### Assessment of guidelines using AGREE II

All appraisals were made using My AGREE PLUS interactive guideline appraisal platform **(**www.agreetrust.org) by 3 reviewers (AS, AH, and MR). Two authors had previous experience with the AGREE II instrument,^6^ and one reviewer (AS) underwent the online AGREE II tutorial before the review (available at: http://www.agreetrust.org/).

The AGREE II is a 23-item tool organized within 6 domains: (1) scope and purpose; (2) stakeholder involvement; (3) rigor of development; (4) clarity of presentation; (5) applicability, and (6) editorial independence. The AGREE II instrument also contains 2 global rating items: (1) overall guideline assessment (that requires the appraiser to make an overall judgement of the practice guideline while considering how they rated the 23 key items) and (2) a question on whether the appraiser would recommend a guideline for use in practice (assessed on a 3-point scale [ie, yes, yes with modification, and no]). All of the AGREE II items and the overall guideline assessment item are assessed using a 7-point Likert agreement scale ranging from 1 (strongly disagree) to 7 (strongly agree). The reviewers discussed all scores that differed by 2 or more points among themselves, until a consensus was reached.

For each item and domain, the score was summed and calculated as a percentage of the maximum possible score for that item/domain using the formula provided by the AGREE II consortium:^4^ [(score obtained – minimum possible score)/(maximum possible score – minimum possible score)] x 100. The possible standardized scores range from 0% (the minimum) to 100% (the maximum).

The AGREE II does not provide a minimum or maximum range for domain score quality to differentiate high- and low-quality guidelines and recommends that it should be done by the reviewer. In agreement with a previous quality appraisal with the AGREE II of the same clinical question carried out by members of the current review group,^1^ a standardized domain score of above 60% for each domain has been chosen as the threshold.

### Statistical analysis and data synthesis

Normality of quality scores was assessed using the Shapiro-Wilk test and based on visual assessment of histograms. Due to the lack of a normal distribution of scores, data are presented as the median followed by the quartiles (upper [Q3] and lower [Q1]) and IQR (interquartile range). Agreement between raters (inter-rater reliability) was analyzed using Fleiss' Kappa and intraclass correlation coefficient (ICC) estimates. The ICC calculation was based on a single rating, absolute agreement, two-way random effects model including a 95% confidence interval (CI). Analysis was conducted in R software, version 3.5.1 (http://cran.r-project.org). by an independent statistician. Although Kendall's W coefficient was pre-specified in the protocol to assess agreement between raters, after consultation with the statistician, it was changed to Fleiss' Kappa that is suitable for analysis of the agreement using ordinal or nominal parameters (either dichotomous or not).^7^

## Results

**Fig. 1:**
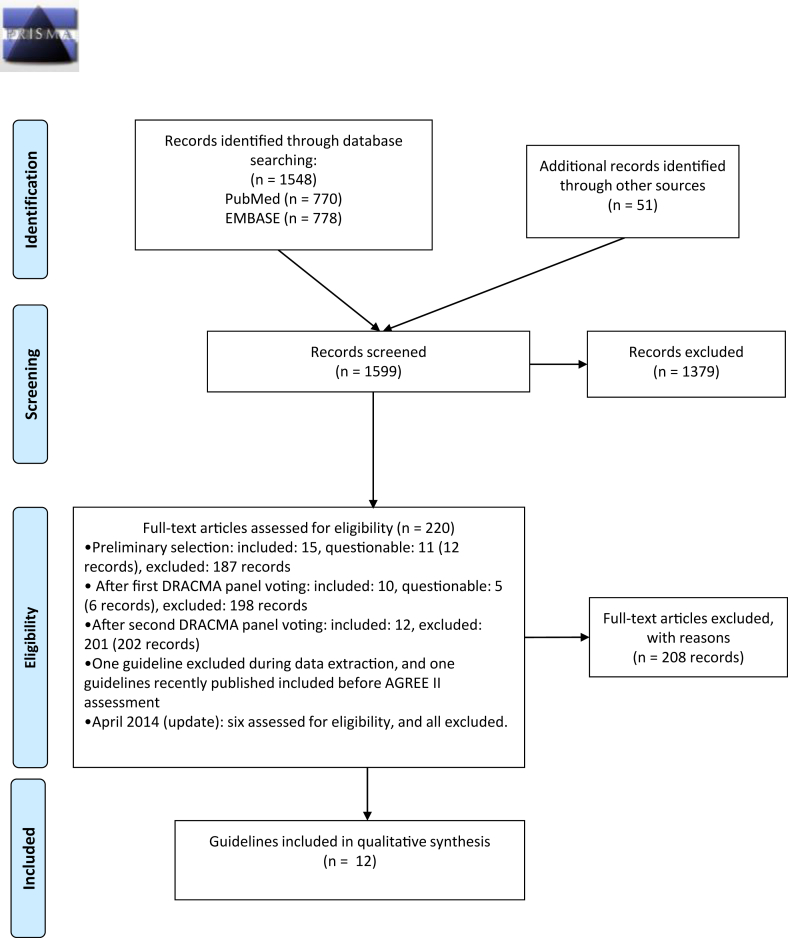
Study selection (PRISMA Flow chart)

For the guideline selection process, see Fig. 1. Excluded guidelines with reasons for exclusion are summarized in Supplementary Table 1.

### Characteristics of included guidelines

We included 12 guidelines (for characteristics, see Table 1). Eight guidelines were developed by national organizations (India, Italy, France, Finland, 2 from Spain, and 2 from the United Kingdom), and 4 by international organizations and the International FPIES Association [I-FPIES] advocacy group; Gastroenterology Committee of the European Society for Paediatric Gastroenterology, Hepatology, and Nutrition [ESPGHAN]; General Practice Infant Feeding Network [GPIFN] and the Milk Allergy in Primary [MAP] Care team; and the World Allergy Organization [WAO] Special Committee on Food Allergy).

**Table 1:** Characteristics of the included guidelines.

1. EWGPAG (Italy, 2010)^8^	
Organization	The Emilia-Romagna Working Group for Paediatric Allergy and for Paediatric Gastroenterology (EWGPAG)
Population	Children, mainly refers to the first year of age
Financial support	Funding not reported.
Conflict of interest	No competing interests have been declared.
2. CNSFP (France, 2018)^9^	
Organization	Committee on Nutrition of the French Society of Paediatrics (CNFSP)
Population	Children
Financial support	Funding not reported.
Conflict of interest	6/12 authors declared to have financial conflict of interest
3. Spanish on non-IgE-mediated CMA (Spain, 2019)^15^	
Organization	Spanish Society of Pediatric Gastroenterology, Hepatology, and Nutrition (SEGHNP)The Spanish Association of Pediatric Primary Care (AEPAP)The Spanish Society of Extra-hospital Paediatrics and Primary Health Care (SEPEAP)The Spanish Society of Pediatric Clinical Immunology, Allergy, and Asthma (SEICAP)
Population	Children
Financial support	Funding not reported.
Conflict of interest	7/11 authors declared to have financial conflict of interest.
4. WAO (international, 2010)^2^	
Organization	The World Allergy Organization (WAO) Special Committee on Food Allergy identified *targeted (and tapped for their expertise), both on the DRACMA panel or as nonsitting reviewers, were allergists, pediatricians (allergists and generalists), gastroenterologists, dermatologists, epidemiologists, methodologists, dieticians, food chemists, and representatives of allergic patient organizations*
Population	All ages, especially young ones
Financial support	The WAO Special Committee on Food Allergy is supported through unrestricted educational grants from various charities and companies that are representative of the food industry: Danone, Heinz, Ordesa, Nestle Nutrition, Dicofarm, and Invest for Children.The content of the Guidelines was developed independently, and the GRADE evaluation of the Guidelines was independently conducted at McMaster University in Hamilton, Ontario, Canada, under Holger Schunemann assisted by Jan Brozek, Enrico Compalati and Luigi Terracciano.
Conflict of interest	Individual conflict of interest not reported.
5. GPIFN and MAP (international, 2019)^10^	
Organization	Members of General Practice Infant Feeding Network (GPIFN) and other infant feeding healthcare leads and the Milk Allergy in Primary (MAP) Care team. Dr Lovis joining them to work alongside representatives from the Cows' Milk Allergy Support group. The current iteration of the MAP guideline has received patient input from members of a large, online CMA community, Cow's Milk Protein Allergy Support, members of the General Practice Infant Feeding Network and other infant feeding healthcare leads, none of whom has any industry ties (UK).
Population	Children, especially infants
Financial support	No funding was received for any aspect of this work.
Conflict of interest	iMAP was developed without any funding or support from industry but 9/12 authors made declarations of interest.
6. ESPGHAN (Europe, 2012)^11^	
Organization	European Society for Paediatric Gastroenterology, Hepatology, and Nutrition (ESPGHAN) Gastroenterology (GI) Committee
Population	Infants and children
Financial support	Funding not reported.
Conflict of interest	11/12 authors declared to have financial conflict of interest.
7. BSACI (United Kingdom, 2014)^17^	
Organization	Standards of Care Committee (SOCC) of the British Society for Allergy and Clinical Immunology (BSACI)
Population	Children and adults
Financial support	Funding not reported.
Conflict of interest	6/7 authors declared to have financial conflict of interest.
8. Spanish on IgE-mediated CMA (SEICAP) (Spain, 2015)^16^	
Organization	Food allergy committee of SEICAP (Spanish Society of Pediatric Allergy, Asthma and Clinical Immunology)
Population	Children and adults
Financial support	Funding not reported.
Conflict of interest	The authors have no conflict of interest to declare.
9. ISPGHAN (Indie, 2020)^12^	
Organization	The pediatric gastroenterology sub-specialty chapter of Indian Academy of Pediatrics (Indian Society of Pediatric Gastroenterology, Hepatology & Nutrition ISPGHAN).*A group of experts.*
Population	Children
Financial support	There was no funding.
Conflict of interest	The authors have no conflict of interest to declare.
10. NICE (United Kingdom, 2019)^18^	
Organization	National Institute for Health and Care Excellence (NICE)
Population	Children. Focused on aged 5 years and younger.These guidelines do not cover the management of cow's milk allergy in older children and adults.
Financial support	Nothing to declare.
Conflict of interest	Nothing to declare.
11. AAAAI and I-FPIES (international, 2017)^13^	
Organization	The Adverse Reactions to Food Committee. American Academy of Allergy, Asthma and Immunology, AAAAI, International FPIES Association advocacy group, I-FPIES
Population	Children
Financial support	This project has been developed in collaboration with The International FPIES (I-FPIES) Association.
Conflict of interest	25/41 authors declared to have potential financial conflict of interest outside of the scope of the guidelines.
12. Finnish guidelines (the Finnish Allergy Programme) (Finland, 2012)^14^	
Organization	The Finnish Allergy Programme 2008–2018.Local Allergy Working Group has been created in different part of Finland (Finland).
Population	Children
Financial support	This work was supported by the European Research Council Advanced Grant 232826 to I.H., the European Commissions 7th Framework Programme under grant agreement 261357, Ministry of Social Welfare and Health, Academy of Finland, Helsinki University Hospital, and the Juselius Foundation.
Conflict of interest	Conflict of interest not reported.

AAAAI, American Academy of Allergy, Asthma and Immunology; AEPAP, Spanish Association of Paediatric Primary Care; BSACI, British Society for Allergy and Clinical Immunology; CNSFP, Committee of Nutrition of the French Society of Paediatrics; ESPGHAN, European Society of Paediatric Gastroenterology, Hepatology and Nutrition; EWGPAG, the Emilia-Romagna Working Group for Paediatric Allergy and that for Paediatric Gastroenterology; GPIFN, General Practice Infant Feeding Network; I-FPIES, International Food Protein-Induced Enterocolitis Syndrome (FPIES) Association; ISPGHAN, Indian Society of Pediatric Gastroenterology, Hepatology and Nutrition; MAP, Milk Allergy in Primary; NICE, National Institute for Health and Care Excellence; SEICAP, Spanish Society of Pediatric Allergy, Asthma and Clinical Immunology; SEGHPN, Spanish Society of Paediatric Gastroenterology, Hepatology, and Nutrition; SEICAP, Spanish Society of Paediatric Clinical Immunology Allergy, and Asthma SEPEAP, Spanish Society of Extra-hospital Paediatrics and Primary Health Care; WAO, World Allergy Organization

Eight guidelines were focused only on children.^8^, ^9^, ^10^, ^11^, ^12^, ^13^, ^14^, ^15^ Two guidelines were not only on the management of CMA in children, but also in adults.^16^^,^^17^ One set of guidelines, although developed with regard to all ages, was focused especially on young ones;^2^ the second was directed mostly at children aged 5 years and younger,^18^ however, older children and adults were also discussed.

Three guidelines were focused on the diagnosis and management of infants with any CMA.^8^^,^^9^^,^^14^ Among 2 Spanish guidelines, one^15^ included recommendations for management of infants only with non-IgE-mediated CMA, and one^16^ for infants only with IgE-mediated CMA. Five guidelines provided recommendations with regard to IgE-mediated and non-IgE-mediated CMA separately.^10^, ^11^, ^12^^,^^17^^,^^18^ One set of guidelines^2^ provided recommendation only for IgE-mediated CMA (and non-IgE-mediated CMA recommendations were in a review). One set of guidelines reported recommendations on the diagnosis and management of infants only with FPIES.^13^ Half of the included guidelines^9^^,^^10^^,^^12^^,^^13^^,^^16^^,^^18^ were published in the last 5 years.

### Quality of included guidelines (the AGREE II quality scores)

Table 2 provides the individual domain scores as well as the overall scores for CMA guidelines assessed using the AGREE II instrument. The scores for each domain varied. Of all the domains, the clarity of presentation domain had the highest median score (92%; Q1-Q3: 81–100%), whereas rigor of development was assessed with the lowest median score (30%; Q1-Q3: 15–67%).

**Table 2:** Domain scores and overall assessment of CMA guidelines using the AGREE II instrument.

Endorsed society of guidelines (country, year)	AGREE II domain scores						Overall score
	1	2	3	4	5	6	
	scope and purpose	stakeholder involvement	rigor of development	clarity of presentation	applicability	editorial independence	
NICE (United Kingdom, 2019)^18^	98%	100%	100%	100%	100%	100%	100%
BSACI (United Kingdom, 2014)^17^	100%	74%	91%	100%	82%	100%	100%
WAO (international, 2010)^2^	100%	100%	97%	100%	89%	58%	100%
AAAAI and I-FPIES (international, 2017)^13^	89%	56%	90%	100%	67%	100%	100%
EWGPAG (Italy, 2010)^8^	89%	83%	32%	94%	68%	75%	78%
Spanish on non-IgE-mediated CMA (SEGHPN, AEPAP, SEPEAP, and SEICAP) (Spain, 2019)^15^	100%	70%	44%	100%	47%	75%	72%
GPIFN and MAP (international, 2019)^10^	70%	85%	28%	81%	69%	100%	50%
ISPGHAN (Indie, 2020)^12^	72%	24%	14%	89%	58%	100%	67%
Spanish on IgE-mediated CMA (SEICAP) (Spain, 2015)^16^	74%	9%	15%	83%	81%	72%	61%
ESPGHAN (Europe, 2012)^11^	69%	22%	20%	81%	63%	75%	61%
CNSFP (France, 2018)^9^	59%	17%	13%	81%	53%	56%	44%
Finnish guidelines (the Finnish Allergy Programme) (Finland, 2012)^14^	22%	15%	4%	50%	14%	53%	17%
**Median**	**82%**	**63%**	**30%**	**92%**	**68%**	**75%**	**70%**
**q1**	70%	21%	15%	81%	57%	69%	58%
**q3**	99%	79%	67%	100%	75%	100%	89%
**IQR**	29%	58%	52%	19%	18%	32%	31%

All individual items for each domain and overall score were assessed using 1–7 point Likert scale. AGREE II domain and overall scores were calculated by summing up the individual scores for all items of each domain/all ratings of the overall quality and calculating as a percentage of the maximum possible score for that domain (where 0% was the minimum, and 100% was the maximum), using the formula provided by the AGREE II consortium^4^: [(score obtained – minimum possible score)/(maximum possible score – minimum possible score)] x 100.

AAAAI, American Academy of Allergy, Asthma and Immunology; AEPAP, Spanish Association of Paediatric Primary Care; BSACI, British Society for Allergy and Clinical Immunology; CNSFP, Committee of Nutrition of the French Society of Paediatrics; ESPGHAN, European Society of Paediatric Gastroenterology, Hepatology and Nutrition; EWGPAG, the Emilia-Romagna Working Group for Paediatric Allergy and that for Paediatric Gastroenterology; GPIFN, General Practice Infant Feeding Network; MAP, Milk Allergy in Primary; I-FPIES, International Food Protein-Induced Enterocolitis Syndrome (FPIES) Association; ISPGHAN, Indian Society of Pediatric Gastroenterology, Hepatology and Nutrition; NICE, National Institute for Health and Care Excellence; SEGHPN, Spanish Society of Paediatric Gastroenterology, Hepatology, and Nutrition; SEICAP, Spanish Society of Paediatric Clinical Immunology Allergy, and Asthma SEPEAP, Spanish Society of Extra-hospital Paediatrics and Primary Health Care; WAO, World Allergy Organization

Inter-rater agreement measured with Fleiss' Kappa varied from 0.552 to 0.730 with the median value across all guidelines of 0.813 (Q1-Q3: 0.7325 to 0.873). ICC absolute agreements varied from 0.574 (95% CI, 0.338 to 0.770) to 0.993 (95% CI, 0.986 to 0.997). For one set of guidelines (National Institute for Health and Care Excellence [NICE]),^18^ there was no variation in responses measured with Fleiss’ Kappa and ICC (100% agreement).

#### Scope and purpose (domain 1)

The median score for the scope and purpose domain was 82% (Q1-Q3: 70–99%) across all guidelines. Three guidelines (British Society for Allergy and Clinical Immunology [BSACI], Spanish on non-IgE-mediated CMA and WAO)^2^^,^^15^^,^^17^ achieved the highest median score (100%), and one set of guidelines (NICE)^18^ achieved a median score equal to 98%. Two guidelines with the lowest ratings achieved median scores for this domain below 60%.^9^^,^^14^ Low scores were mainly due to a lack of proper reporting, including a non-specified overall objective and a poor description of a target population.

#### Stakeholder involvement (domain 2)

For the stakeholder involvement domain, the median score was 63% (Q1-Q3: 21–79%). Two guidelines (NICE, WAO)^2^^,^^18^ achieved the maximum median score (100%). Six guidelines^9^^,^^11^, ^12^, ^13^, ^14^^,^^16^ did not achieve a median score of 60% for this domain. The main reason for such low scores was a lack of assessment of the views and preferences of the target population (patient, public, etc.).

#### Rigor of development (domain 3)

For this domain, the median score was 30% (Q1-Q3: 15–67%). The highest median score (100%) was achieved only by 1 set of guidelines (NICE).^18^ The median score for 8 guidelines^8^, ^9^, ^10^, ^11^, ^12^^,^^14^, ^15^, ^16^ did not exceed 44%. The main reasons for low scores for this domain were unclear description of strengths and limitations of the body of evidence and a lack of reporting of the procedures for updating the guidelines.

#### Clarity of presentation (domain 4)

The median score for this domain was 92% (Q1-Q3: 81–100%). Five guidelines (AAAAI and I-FPIES, BSACI, NICE, Spanish on non-IgE-mediated CMA and WAO)^2^^,^^13^^,^^15^^,^^17^^,^^18^ achieved the highest median score (100%). Only 1 set of guidelines^14^ did not exceed a median score of 60%, in which the main reason for the low score was the lack of easily identifiable key recommendations.

#### Applicability (domain 5)

The median score for this domain was 68% (Q1-Q3: 57–75%). Only 1 set of guidelines (NICE)^18^ achieved the highest possible score (100%). The median score for 4 guidelines^9^^,^^12^^,^^14^^,^^15^ did not exceed 60% (Q1-Q3: 14–58%). The main limitation was a lack of or not clearly described facilitators and barriers for application of these guidelines.

#### Editorial independence (domain 6)

For this domain, the median score was 75% (Q1-Q3: 69–100%). Five guidelines (AAAAI and I-FPIES, BSACI, GPIFN and MAP, Indian Society of Pediatric Gastroenterology [ISPGHAN], and NICE)^10^^,^^12^^,^^13^^,^^17^^,^^18^ achieved the highest possible median score (100%). Three guidelines^2^^,^^9^^,^^14^ had a median score below 60%. The low score was mainly due to the lack of reporting of the competing interests of the guideline development group members.

#### Overall quality score

The median overall score was 70% (Q1-Q3: 58–89%). The maximum possible overall score was 100% and it was achieved by four guidelines (AAAAI and I-FPIES, BSACI, NICE, WAO).^2^^,^^13^^,^^17^^,^^18^ For 3 guidelines,^9^^,^^10^^,^^14^ the median overall score did not achieve 60% (Q1-Q3: 17–50%).

### Summary of recommendations

Table 3 provides a summary of specific recommendations listed separately for each recommendation or clinical indication.

**Table 3:** Summary of specific recommendations.

Diagnosis of CMA	
EWPGAG 2010^8^	Any CMA
WAO 2010^2^	Only for IgE-mediated CMA (non-IgE-mediated in a review)
Finnish guidelines 2012^14^	Any CMA
ESPGHAN 2012^11^	Separately for IgE-mediated and non-IgE-mediated CMA
BSACI 2014^17^	Separately for IgE-mediated and non-IgE-mediated CMA
SEICAP 2015^16^	Only for IgE-mediated CMA
AAAAI and I-FPIES 2017^13^	Only for CM-FPIES
CNSFP 2018^9^	Not reported
SEGHPN, AEPAP, SEPEAP, and SEICAP 2019^15^	Only for non-IgE-mediated CMA
GPIFN and MAP 2019^10^	Separately for IgE-mediated (only diagnosis) and non-IgE-mediated CMA
NICE 2019^18^	Separately for IgE-mediated and non-IgE-mediated CMA
ISPGHAN 2020^12^	Separately for IgE-mediated and non-IgE-mediated CMA

Clinical history and physical examination to establish suspicion of CMA	
EWPGAG 2010^8^	Recommendation for a collection of detailed history of symptoms to establish suspicion of CMA.
WAO 2010^2^	*Not as official recommendation.*
ESPGHAN 2012^11^	Recommendation for a collection of detailed history of symptoms and physical examination.
BSACI 2014^17^	Recommendation for a collection of detailed history of symptoms (including severity evaluation).
SEICAP 2015^16^	Recommendation for a collection of detailed history of symptoms and physical examination.
AAAAI and I-FPIES 2017^13^	Recommendation for a collection of clinical history of typical signs and symptoms for both acute and chronic FPIES, and to consider a broad differential for a patient with acute vomiting in a diagnosis of FPIES.
SEGHPN, AEPAP, SEPEAP, and SEICAP 2019^15^	Recommendation for a collection of detailed history of symptoms, physical examination, growth assessment, and feeding history.
GPIFN and MAP 2019^10^	Recommendation for a specifically allergy-focused clinical history and physical examination.
NICE 2019^18^	Recommendation for a specifically allergy-focused clinical history and physical examination, including: nutritional status and growth (weight, length/height, and calculation of BMI), any signs of a clinical reaction, or comorbid conditions such as atopic eczema, asthma, and/or allergic rhinitis, or suggesting an alternative diagnosis.
ISPGHAN 2020^12^	Recommendation for a collection of detailed history of symptoms and physical examination.
Other guidelines^9^^,^^14^	Not reported.

Elimination-reintroduction	
EWPGAG 2010^8^	Recommendation for use of CMP elimination diet and, in case of resolution of symptoms, confirmation with OFC. If IgE-mediated CMA, supervised challenge in minority of cases.Not recommended in: - exclusively breastfed infants with bloody stools (proctocolitis), - with suspected reaction to CMA and mild symptoms, - with mild AD and negative history for CM reactions. Children with any severe symptoms should be referred to a specialized center*.*
WAO 2010^2^	Suspected IgE-mediated CMA: In settings in which an OFC is not a requirement, in patients with an average pretest probability of IgE-mediated CMA, suggestion for use of OFC with CM as the only test without measuring milk sIgE levels as a triage or add-on test.
Finnish guidelines 2012^14^	Recommendation for use of elimination diet with no milk or egg and, in case of resolution of symptoms, referral to a specialist who will supervise an OFC.
ESPGHAN 2012^11^	Recommendation for use of CMP elimination diet and, in case of resolution of symptoms, confirmation with standardized OFC (not if clear immediate type reaction or anaphylaxis).
BSACI 2014^17^	Recommendation for use of CMP elimination diet and, in case of resolution of symptoms, confirmation with OFC (in IgE-mediated CMA; if diagnostic uncertainty [conflict between the history and diagnostic tests], in non-IgE-mediated CMA, a gold standard).
SEICAP 2015^16^	Recommendation for controlled oral provocation: (1) if negative SPT and/or sIgE, (2) in patients with chronic symptoms such as AD and urticaria, and positive allergy test,
AAAAI and I-FPIES 2017^13^	Diagnosis primarily based on a clinical history of typical characteristics signs and symptoms with improvement after withdrawal of the suspected trigger food.Recommendation for exclusion of other potential causes and use of OFC only if the unclear history and a favorable risk/benefit ratio.In patients with suspected chronic FPIES, who become asymptomatic and maintain normal growth when the trigger food is eliminated from the diet, subsequent reintroduction of the trigger food induces acute FPIES symptoms within 1–4 h.
SEGHPN, AEPAP, SEPEAP, and SEICAP 2019^15^	Recommendation for use of CMP elimination diet and, in case of resolution of symptoms, confirmation with OFC (depends if severe cases, suspected FPIES, or possible IgE-mechanism).If severe cases or suspected FPIES, or no improvement on elimination diet, referral to specialist.If CMA is still suspected despite of lack of response to diet, a suggestion for the exclusion of other foods (ie, soy protein and egg), and in case of formula-fed infants to switch to another EHF or hydrolyzed rice formula.
GPIFN and MAP 2019^10^	Mild to moderate IgE-mediated CMA: Recommendation for use of CMP elimination diet and, in case of resolution of symptoms, confirmation with OFC (mostly in non-IgE-mediated CMA).Mild to moderate non-IgE-mediated CMA: Re-introduction of CM at home. CMA is confirmed only if symptom improves after return to elimination diet after home re-introduction.Severe non-IgE-mediated or mild to moderate IgE-mediated CMA: Referral to local pediatric allergy service (also if no improvement despite elimination diet and CMA still suspected) and dietitian.Severe IgE-mediated CMA (anaphylaxis): Emergency treatment and admission.
NICE 2019^18^	Recommendation for use of CMP elimination diet and, in case of resolution of symptoms, confirmation with OFC (home reintroduction). CMA confirmed only if symptom improves after return to elimination diet after OFC. If CMA still suspected despite a lack of response to diet, referral to specialist for advice to eliminate other foods (ie, soy protein or egg), in formula-fed infants switching EHF to AAF.Recommendation for use of OFC to confirm diagnosis of IgE-mediated CMA if inconsistency between the history and diagnostic tests. Referral to a specialist allergy clinic and/or pediatric dietitian with the urgency depending on clinical judgement (indications in guidelines).
ISPGHAN 2020^12^	Recommendation for use of CMP elimination diet and, in case of resolution of symptoms, OFC.
CNSFP 2018^9^	Not reported.

Duration of diagnostic elimination diet	
EWPGAG 2010^8^	2–4 week period (4 weeks for gastrointestinal symptoms), 10 days if enterocolitis syndrome, 1–3 weeks for enteropathy, 6 weeks for eosinophilic esophagogastroenteropathy.
WAO 2010^2^	*Not as official recommendation.*
Finnish guidelines 2012^14^	1–2 weeks if skin symptoms, 2–4 weeks if gastrointestinal symptoms.
ESPGHAN 2012^11^	1–2 weeks if early and late reactions (ie, vomiting, atopic eczema), 2–4 week if gastrointestinal symptoms (ie, diarrhea, constipation). If the history suggests an immediate reaction, only 3 to 6 days. If delayed reactions are suspected (eg, allergic proctocolitis), then up to 14 days.
BSACI 2014^17^	At least 6 weeks in infants with eczema.
SEICAP 2015^16^	No longer than 2–3 weeks.
SEGHPN, AEPAP, SEPEAP, and SEICAP 2019^15^	2–4 weeks depending on symptoms and severity: 1–5 days in acute forms (acute FPIES, vomiting), 1–2 weeks for eczema/gastrointestinal bleeding, 2–4 weeks in cases of constipation, diarrhea, growth faltering.
GPIFN and MAP 2019^10^	2–4 weeks.
NICE 2019^18^	2–4 weeks.
ISPGHAN 2020^12^	From 3 to 5 days (IgE-mediated CMA) to 2–4 weeks (other than IgE-mediated, max 4 weeks). 1–2 week for most, 2–4 week for chronic symptoms. [Differences in the paper: The maternal elimination diet is maintained for 3 to 6 days in those with IgE-mediated allergy, while in non-IgE mediated it is two weeks in those without atopy, and 4 weeks in those with atopic dermatitis or allergic]
Other guidelines^9^^,^^13^	Not reported.

Settings of OFC	
EWPGAG 2010^8^	- Under medical supervision, in a setting with emergency facilities, especially in case of positive SPT or sIgE to CM and infants at risk of an immediate reaction. - Open or blinded challenge. - Recommendation against OFC in children with immediate reactions or late gastrointestinal reactions with anemia, poor growth, or hypoalbuminemia if causative role of CM is clear.
WAO 2010^2^	- Under the supervision of a specialist. Except delayed allergic reaction (chronic diarrhea, colitis, allergic proctocolitis, gastroesophageal reflux) without sIgE, OFC in hospital settings. - Double-blind placebo-controlled food challenge method of choice in research and delayed reaction settings, and with uncertain outcome. In other cases, open OFC.
Finnish guidelines 2012^14^	Under specialist supervision.
ESPGHAN 2012^11^	Standardized OFC under medical supervision (inpatient or outpatient settings).
BSACI 2014^17^	- In hospital (attached protocol). - Challenge food is baked or fresh milk, reactions to baked milk are less likely to be severe, and tolerance to baked milk is developed earlier than to fresh milk (home baked CM reintroduction).
SEICAP 2015^16^	- Under medical supervision. - Double-blind placebo-controlled food challenge is the gold standard (reserved for research), however, open provocation or simple-blinding test acceptable in daily practice. - Recommendation against if a positive SPT/sIgE for milk with a recent clinical episode (within the last 3 months).
AAAAI and I-FPIES 2017^13^	- In medically supervised setting with access to rapid fluid resuscitation and prolonged observation. - Recommendation against the home OFCs to a food suspected of triggering FPIES given the potential for severe reactions. - It is generally recommended not to exceed a total of 3 g of protein or 10 g of total food (100 mL of liquid) for an initial feeding (which aims to OFC if there approximate a serving size) and observe the patient for 4 to 6 h.
SEGHPN, AEPAP, SEPEAP, and SEICAP 2019^15^	- Home reintroduction, only if there is confirmed lack of IgE sensitization. - Under supervision of the pediatrician in patients with proctocolitis, GOR, colic, constipation and other mild gastrointestinal symptoms. - In hospital, in cases of the immediate reactions, severe atopic dermatitis, FPIES, moderate to severe enteropathy, in whom an IgE-mediated mechanism is suspected. - The period of observation after reintroduction of CMP should be of at least 2 weeks and of up to 4 weeks, especially in cases with constipation or enteropathy.
GPIFN and MAP 2019^10^	- Mild to moderate IgE mediated CMA: some may need OFC in hospital setting - Mild to moderate non-IgE mediated CMA: home reintroduction with CMP (return to regular maternal or infant's diet or standard CM formula)
NICE 2019^18^	- Non-IgE-mediated CMA: home reintroduction with CM (return to regular maternal or infant's diet, or standard CM formula) - IgE-mediated CMA: the administration of increasing quantities of baked or fresh CM under medical supervision, starting with direct mucosal exposure (allergen contact with the lips) and then titrated oral ingestion as tolerated. The rate of dose escalation, the time interval between doses, and observation period after the challenge depends on the individual child's presentation.
ISPGHAN 2020^12^	- Under medical supervision. - Double-blind placebo-controlled food challenge is the gold standard; however, mostly open challenge is performed. - Not recommended if patient with severe anaphylaxis.
CNSFP 2018^9^	Not reported.

Cow's milk specific IgE (sIgE) and skin prick tests (SPT)	
EWPGAG 2010^8^	Infant with immediate and late reactions: Referral to a specialized clinic for SPT and/or sIgE.
WAO 2010^2^	- In setting where OFC is not a requirement and high pretest probability of IgE-mediated CMA, and SPT with a cut-off value of ≥3 mm – no OFC; or low patient pretest probability of CMA if SPT below cut-off value – no OFC. - In setting where OFC is not a requirement and high pretest probability of IgE-mediated CMA, sIgE with a threshold of 0.7 IU/L – if positive, no OFC. If low pretest probability of IgE-mediated CMA, sIgE with a cut-off value of ≥0.35 IU/L – if negative, no OFC.
ESPGHAN 2012^11^	- Recommendation for sIgE and elimination diet in infants with presence of anaphylaxis or clear immediate type reaction (if negative result, the OFC). - The presence of CMP-sIgE and/or a positive SPT to CM indicates IgE-mediated CMA; however, results must be interpreted in the context of medical history and OFC. - Combination of the sIgE and SPT not necessary.
BSACI 2014^17^	The clinical diagnosis of IgE-mediated CMA based on combination of typically presented symptoms soon after ingestion of CM and evidence of sensitization (sIgE and/or SPT tests). SPT, if IgE-mediated CMA suspected. If below 3 mm, to repeat or consider sIgE. If SPT weal diameter 2–4 mm, to consider OFC. A SPT weal size ≥5 mm (≥2 mm in younger infants) is strongly predictive of CMA.
SEICAP 2015^16^	SPT and/or sIgE recommended. If negative, to reconsider diagnosis, and controlled OFC. If positive SPT and/or sIgE, but not recent episode, an OFC. For sIgE, a positivity cut-off value is 0.35 kUA/L. For SPT, positivity cut off is at least 3 mm SPT size wheal.
AAAAI and I-FPIES 2017^13^	Recommendation against routinely performed testing for food sIgE to identify food triggers of FPIES (non-IgE-mediated process). sIgE may be considered in patients with CM-FPIES only with certain comorbid conditions as IgE-mediated allergies, AD or respiratory allergic disorders.
SEGHPN, AEPAP, SEPEAP, and SEICAP 2019^15^	Recommendation for use of sIgE or SPT in patients with severe AD and/or FPIES before OFC (if positive result, the OFC following the protocol of IgE-mediated reaction).Recommendation against use of SPT or sIgE, if any doubt about an IgE mechanism.
GPIFN and MAP 2019^10^	Suspected IgE-mediated CMA: IgE testing, particularly in children with eczema after a prolonged period of avoidance.
NICE 2019^18^	Suspected IgE-mediated CMA: SPT or sIgE recommended. If results not corresponding with history, or the equivocal history, supervised OFC recommended.
ISPGHAN 2020^12^	- sIgE and SPT not useful in diagnosis of non-IgE-mediated CMA. - SPT can be considered in IgE-mediated CMA: a positive test do not confirm allergy, a negative SPT rules out IgE-mediated CMA. - Acute/life threatening symptoms (ie, stridor, wheeze, angioedema and anaphylaxis): if CMP-sIgE positive and resolution of symptom with an elimination diet, the OFC may be delayed by a year.
Other guidelines^8^^,^^9^^,^^14^	Not reported.

Not recommended tests	
WAO 2010^2^	Routine use of molecular-component resolved diagnostics. Allergen microarrays only in research.
ESPGHAN 2012^11^	Atopy patch testing, determination of total IgE, the ratio of sIgE to total IgE, determination of IgG antibodies or IgG subclass antibodies against CMP, applied kinesiology (muscle strength testing) and hair analysis (assessing mineral content), facial thermography, gastric juice analysis, provocation neutralization, cytotoxicity assay, electrodermal testing, intradermal testing (a risk of systemic allergic reaction in highly sensitized individuals).Basophil/histamine release/activation, lymphocyte stimulation, mediator release assay, endoscoping allergen provocation recommended in research, but not in clinical practice.
BSACI 2014^17^	Hair analysis, kinesiology, iridology, electrodermal testing (Vega), lymphocyte stimulation tests and food-specific IgG and IgG4, histamine, tryptase, and chymase assays.Recommendation against routine use of molecular-component resolved diagnostics
SEICAP 2015^16^	Intradermal tests, the patch tests with commercial antigens, IgG and its components, basophil activation testing or microarray techniques.
SEGHPN, AEPAP, SEPEAP, and SEICAP 2019^15^	Routine radiographic testing if CM-FPIES suspected, and routine performance of laboratory tests, and routine endoscopy, unless the diagnosis is uncertain, or patient do not respond to elimination diet with endoscopy based on judgement of gastroenterologist.Recommendation against atopy patch testing.
NICE 2019^18^	Atopy patch testing, serum-specific immunoglobulin (Ig)G testing, applied kinesiology (muscle strength testing), hair analysis (assessing mineral content) and vega testing (electroacupuncture devices).
ISPGHAN 2020^12^	The cow's milk-related symptom score (CoMiSS).
Other guidelines^8^, ^9^, ^10^^,^^13^^,^^14^	Not reported.

Other recommended tests	
AAAAI and I-FPIES 2017^13^	- Assessment of chemistry or blood count in the acute setting in differential diagnosis of FPIES. - A work-up to rule out other gastrointestinal diseases (eg, enteropathy, eosinophilic esophagitis, very early onset inflammatory bowel disease, primary immunodeficiency syndromes) resulting in symptoms that overlap with FPIES.
ISPGHAN 2020^12^	Sigmoidoscopy and rectal biopsy in patients with only gastrointestinal manifestation (enterocolitis presentation).
Other guidelines^2^^,^^8^, ^9^, ^10^, ^11^^,^^14^, ^15^, ^16^, ^17^, ^18^	Not reported.

Breastfeeding	
EWPGAG 2010^8^	- Breast-fed infants: a diagnostic maternal diet without CM not recommended for mild symptoms. Infants with bloody stools (proctocolitis): recommendation against the maternal diet without egg and CM. - Elimination of CMP, eggs, and other foods recommended in infants with moderate-severe symptoms only with history of unequivocal reaction. - Confirmed non-IgE CMA (moderate-severe symptoms): the maternal CM elimination diet with supplemental intake of calcium. If the insufficient volume of breast milk, EHF or SF formula (if > 6 months). If no symptoms after the reintroduction of CM in mother's diet, the excluded foods introduced one by one in the diet.
WAO 2010^2^	*Not reported as a recommendation*. - Breast-fed infants: continuation of breast-feeding while avoiding dairy products. - Supplementation: calcium (1000 mg/day divided into several doses) while after a milk-free diet. - Fully breast-fed children more than 2 years: no need to substitute CM if an adequate supply of calcium (600–800 mg/day).
Finnish guidelines 2012^14^	Breastfeeding mothers and to children eating solid foods: a diet eliminating CMP or egg.
ESPGHAN 2012^11^	- Recommendation for continuation of breastfeeding with the maternal CMP-free diet. - Supplementation: calcium supplements (ie, 1000 mg/day spread across the day). - Referral to dietitian. - If there is no improvement: child should be further evaluated. - CMA confirmed: continuation of breastfeeding while maintaining a CMP-free diet (referral to dietitian and supplementation as above) - Symptoms recur on breast milk despite a strict maternal CMP-free diet: further elimination of other highly allergenic foods or weaning from breast milk to a hypoallergenic formula. - The first feeding with CM–based formula in a breast-fed infant causes symptoms: return to exclusive breast-feeding without any elimination in the maternal diet.
BSACI 2014^17^	*Not reported as recommendation.* - Continuation of breastfeeding with maternal CMP elimination diet only if infant is symptomatic. Assessment of mother's need for calcium and vitamin D supplementation. - All breastfed infants over 6 months vitamin D supplementation in the form of vitamin drops.
SEICAP 2015^16^	- Exclusively breastfed infants: recommendation for continuation of breastfeeding with maternal milk and dairy product exclusion diet elimination diet. - Only when breastfeeding not possible: SF, EHF based on CMP, partially hydrolyzed formulae based on rice, or AAF started or added. - Recommendation against maternal elimination diet in infants with atopic dermatitis. - Supplementation: Ca (1000 mg per day). - Infants with mixed feeding: If breastfed without problems and develops symptoms with the introduction of adapted CM formulas, breastfeeding continued without the need for the maternal exclusion diet.
AAAAI and I-FPIES 2017^13^	Recommendation for dietary elimination of the trigger food(s) in the primary management of FPIES.Recommendation against routine maternal dietary elimination of offending triggers while breast-feeding if the infant is thriving and remains asymptomatic.
CNSFP 2018^9^	*Not reported as recommendation.* - In breastfed infants, maternal elimination diet without milk and dairy products. - Supplementation: calcium and vitamin D.
SEGHPN, AEPAP, SEPEAP, and SEICAP 2019^15^	- Exclusively breastfed children: continuation of breastfeeding with CMP-free maternal diet. - Persistence of symptoms despite adequate adherence to the CMP-free diet: to consider the exclusion of other potential food trigger (ie, soy and/or egg). - In mixed-fed infants: if the onset of symptoms coincides with the introduction of formula feeds, return to exclusive breastfeeding (maternal elimination diet mostly not necessary). - Supplementation with calcium (1 g/day) and vitamin D (600 IU/day).
GPIFN and MAP 2019^10^	Suspected IgE-mediated and non-IgE-mediated CMA: - Exclusively breastfeeding mother: if symptomatic on breastfeeding only, trial exclusion of all CMP from her own diet. - Mixed-fed infant: revert to exclusive breastfeeding. If infants asymptomatic on exclusive breastfeeding, recommendation against maternal elimination diet. - Infants with severe AD or more severe gut symptoms: consider seeking specialist advice to also exclude soy protein/egg. - No clear improvement, but CMA still suspected: referral to local pediatric allergy service and to consider exclusion of other maternal foods (ie, soy, egg, only with specialist advice). - Supplementation: calcium and vitamin D following local guidelines. - Referral to dietitian. Treatment of non-IgE CMA (mild to moderate): strict adherence to CM-free diet for the mother/infant until the child is 9–12 month and for at least 6 months with support of dietitian.
NICE 2019^18^	- Exclusively breastfed infants: recommendation for continuation of breastfeeding with maternal elimination diet without CMP. - Mixed-fed infant: revert to exclusive breastfeeding. - Infants asymptomatic on exclusive breastfeeding: recommendation against maternal elimination diet. - Infants with severe non-IgE-mediated allergy and/or AD: consider seeking specialist advice to also exclude soy protein and egg. - Supplementation: calcium and vitamin D according to local protocols. Treatment of non-IgE CMA (mild to moderate), strict adherence to CM-free diet for the mother/infant until the child is 9–12 month and for at least 6 months.
ISPGHAN 2020^12^	Recommendation for continuation of breastfeeding with maternal CMP elimination diet.Supplementation: calcium (1000 mg per day in divided doses).

Extensively hydrolyzed formula for CMAEHWF and EHCF were not discussed separately in any guidelines.	
EWPGAG 2010^8^	- Children <12 months and in older children with severe gastrointestinal symptoms: EHF or AAF. - Children >12 months with anaphylaxis: CM substitutes not always required. - Severe symptoms: EHF or AAF in formula-fed children; if poor growth, anemia, or hypoalbuminemia, AAF for days to 6 week (to switch to EHF). - Mild-moderate symptoms: SF (if older than 6 months of age and no gastrointestinal symptoms) or EHF or AAF. EHF and SF started only under medical supervision. AAF for 2 weeks and then switched to SF or EHF.
WAO 2010^2^	IgE-mediated CMA at low risk of anaphylactic reactions (no prior history of anaphylaxis or currently on EHF): EHFs suggested over AAF, and rather than SF, and extensively hydrolyzed rice formula.
Finnish guidelines 2012^14^	Children under 6 months: EHF. Children over 6 months: either hydrolysate or soy milk.
ESPGHAN 2012^11^	- Formula-fed infants: EHF with proven efficacy usually a first-line choice. Choice of formula depends mostly on the patient age and the other food allergies. - Confirmed CMA: the continuation of elimination diet for at least 6 months or until 9 to 12 months of age. - Infants/children with severe immediate IgE-mediated CMA: elimination diet for 12 or even 18 months before re-challenge after repeated testing for sIgE. The choice of depends on residual allergenic potential, formula composition, costs, availability, infant's acceptance, and clinical data of the formula efficacy. - Infants with enteropathy, diarrhea, and lactose intolerance: a lactose-free EHF as first-line. - Non–breast-fed infants: avoidance of CM–based formula and supplementary foods containing CMP or other unmodified animal milk proteins (eg, goat's milk, sheep's milk)
BSACI 2014^17^	The choice of CM substitute depends on the age of the child, the severity of the allergy, and the nutritional composition of the substitute (a risk of faltering growth and specific nutritional deficiencies).
SEICAP 2015^16^	- Mixed or formula-fed infant: a substitution formula with demonstrated efficacy in CMA. - Symptoms after the intake of EHF: switched to a different EHF or to AAF. - Coexisting secondary lactose intolerance, particularly in infants suffering important digestive alterations with enteropathy and diarrhea: evaluation of lactose-free diet. - Patients extremely sensitive to CMP with positive skin tests with casein hydrolysates: controlled exposure testing with the hydrolysate to check tolerance before introduction; not necessary with products from other sources (rice, soy) or elemental AAF.
AAAAI and I-FPIES 2017^13^	Formula-fed infants or infants who can no longer breastfeed diagnosed with CM-FPIES: a hypoallergenic formula recommended.
CNSFP 2018^9^	A formula with proven safety and suitability in children with CMA should be favored. The efficacy of formulas available in most industrialized countries not always proven by a clinical trial.In a review: In non-breastfed infants: EHFs as the first option.
SEGHPN, AEPAP, SEPEAP, and SEICAP 2019^15^	Formula-fed infants with mild to moderate non-IgE-mediated CMA: casein- or whey-EHF as the first-line choice.In a review: EHFs with medium chain triglycerides should be considered in infants with growth faltering, including formulas containing lactose if lactose intolerance is not suspected.
GPIFN and MAP 2019^10^	Formula-fed or mixed-fed infants: - Mild to Moderate IgE-mediated CMA: If mother unable to revert to fully breastfeeding, EHF as first choice. If diagnosis confirmed (by IgE testing or a supervised challenged in a minority of cases): follow-up with serial IgE testing and later planned challenge to test for acquired tolerance. Dietetic referral required. - Mild to moderate non-IgE-mediated CMA: if mother unable to revert to fully breastfeeding, EHF. - Severe non-IgE-mediated CMA: if mother unable to revert to fully breastfeeding, AAF. Infant asymptomatic on breastfeeding alone: do not exclude CM from maternal diet. Urgent referral to local pediatric allergy service and dietetic referral. - Exclusively breastfed infants with confirmed mild to moderate CMA and need of top-up/supplemental formula: EHF.
NICE 2019^18^	- Infants with suspected non-IgE mediated or IgE-mediated CMA who are formula-fed or mixed-fed, and the mother is unable to return to exclusive breastfeeding: EHF, usually used as first-line (whey or casein-based). - Partially hydrolyzed formulas: not recommended. - Lactose-free formulas not recommended in suspected or confirmed CMA. - The choice of CM substitute should take into account the child's age, growth, severity of symptoms, and nutritional composition. A referral to pediatric dietitian for consideration.
ISPGHAN 2020^12^	Formula-fed infants with mild to moderate IgE or non-IgE-mediated CMA: EHF the first choice (and elimination of all sources of CMP).Infants <6 months of age with mild to moderate reaction: EHF with proven efficacy recommended.Older children: elimination of all forms of milk and milk products.

Modified extensively hydrolyzed formula for CMA (supplemented with pro-, pre- and/or postbiotics)	
ESPGHAN 2012^11^	No evidence of role of probiotics and prebiotics in the treatment of CMA.
SEICAP 2015^16^	A controversy as to whether supplementing EHF with certain probiotics accelerates the acquisition of tolerance.
SEGHPN, AEPAP, SEPEAP, and SEICAP 2019^15^	No sufficient evidence to recommend the routine use of formulas enriched with prebiotic and/or probiotics in the management of children with CMA.
Other guidelines^2^^,^^8^, ^9^, ^10^^,^^12^, ^13^, ^14^^,^^17^	Not reported.

Amino acid formula for CMA (supplemented with pro-, pre- and/or postbiotics)	
EWPGAG 2010^8^	Children with gastrointestinal reactions and anemia, poor growth, or hypoalbuminemia: AAF as first line and then switched to EHF.
WAO 2010^2^	Children with IgE-mediated CMA at high risk of anaphylactic reactions (prior history of anaphylaxis and currently not using EHF): suggested AAF rather than EHF.
Finnish guidelines 2012^14^	Recommendation against immediate transfer to AAF.
ESPGHAN 2012^11^	Infants with extremely severe or life-threatening symptoms or reacting to EHF: AAF may be considered as the first choice.No improvement within 2 weeks on elimination diet (EHF) or infants with significant gastrointestinal symptoms with no improvement using EHF or SF: trial of AAF before CMA is ruled out.Suspected multiple food allergies in highly atopic children or in cases of eosinophilic disorders of the digestive tract: AAF before OFC.Infants with severe anaphylactic reactions or with severe enteropathy indicated by hypoproteinemia and faltering growth: AAF may be considered a first-line treatment despite limited evidence.No improvement on AAF: CMA may be ruled out.
BSACI 2014^17^	AAF for infants with: (1) multiple food allergies, (2) severe CMA, (3) allergic symptoms or severe atopic eczema when exclusively breastfed, (4) severe forms of non-IgE-mediated CMA such as eosinophilic esophagitis, enteropathies, and FPIES, (5) faltering growth and (6) reacting to or refusing to take EHF at nutritional risk.Infants (who meet the criteria for an amino acid milk) require additional energy, Ca, and iron or a flavored product: amino acid follow-on formulas.
SEICAP 2015^16^	AAFs used in cases of serious anaphylactic manifestations and maintained until exposure testing to EHF.AAF considered when EHFs are rejected due to palatability problems.
AAAAI and I-FPIES 2017^13^	*Not reported as recommendation.*
CNSFP 2018^9^	*Not reported as recommendation.*
SEGHPN, AEPAP, SEPEAP, and SEICAP 2019^15^	AAFs: the first-line treatment in severe cases of enteropathy or FPIES, also recommended in no response to treatment with EHF (casein or whey).
GPIFN and MAP 2019^10^	Mild to moderate non-IgE-mediated CMA:No clear improvement after formula fed or ‘Mixed Feeding’: strict CMP-free diet, but CMA still suspected: consideration of AAF and referral to local pediatric allergy service.If top-up/supplement formula feeds needed and EHF is not clinically tolerated: AAF.If formula-fed or mixed-fed with severe symptoms and mother unable to revert to fully breastfeeding, trial of AAF and refer onwards to specialist care.
NICE 2019^18^	AAFs should be reserved for children: (1) with severe symptoms of IgE- or non-IgE-mediated allergy or a history of anaphylaxis, (2) who cannot tolerate or have ongoing symptoms with EHFs, (3) whose symptoms do not respond to maternal avoidance of CM, or have symptoms while exclusively breastfeeding.
ISPGHAN 2020^12^	Children with soy protein allergy, or allergy to other components of the EHF that has been used during milk restriction, or infants with multiple food allergies (such as egg, wheat, soy, nuts, sea fish): AAF.The diagnosis is reasonably certain with no improvement within 2 weeks of EHF,: AAF before CMA is ruled out.Infants who are sick or have severe or life-threatening symptoms: AAF as the first choice rather than EHF.IgE-mediated CMA: No response to EHFs: AAF. Severe allergy that requires hospitalization: AAF.

Plant-based formula (ie, soya-based, rice-based) for CMARice formula	
EWPGAG 2010^8^	A choice in selected cases taking into consideration the taste and the cost.
WAO 2010^2^	In children with IgE-mediated CMA: EHF rather than extensively hydrolyzed rice formula.
ESPGHAN 2012^11^	Hydrolyzed rice formula (partially or extensively hydrolyzed formula) may be considered in infants refusing or not tolerating an EHF based on CMP, or in vegan families.
SEICAP 2015^16^	Partial rice hydrolysate (long-term nutritional studies are lacking) is an option.Hydrolyzed rice protein formula has evidence of safety and nutritional suitability.
CNSFP 2018^9^	*Not reported as recommendation.*
SEGHPN, AEPAP, SEPEAP, and SEICAP 2019^15^	Hydrolyzed rice protein formulas: at any age, an alternative to patients that refuse or do not respond to casein or whey EHF.
Other guidelines^10^^,^^12^, ^13^, ^14^^,^^17^^,^^18^	Not reported.

Soy formula	
EWPGAG 2010^8^	Infants <6 months of age with allergic symptoms and in those with late gastrointestinal symptoms: not recommended
WAO 2010^2^	Children with IgE-mediated CMA: EHF rather than SF.
Finnish guidelines 2012^14^	Recommendation for use of either hydrolysate or soy milk for children over 6 months.
ESPGHAN 2012^11^	EHF or AAF (if EHF not tolerated) preferable over SF in infants with CMA. SF may be considered: - in an infant with CMA older than 6 months if EHF not accepted or tolerated, or too expensive, - or if strong parental preferences (ie, vegan diet).
BSACI 2014^17^	- Not the first line choice of substitute milk for infants <6 months old with CMA. - If hydrolysates not tolerated, AAF. - To consider in infants after 6 months of age because of lower cost or better palatability, after assessment of tolerance to soy protein.
SEICAP 2015^16^	Infants over 6 months of age: may be used.The recommendation against use of SF in infants under 6 months of age (not adequate from the nutritional perspective), and in situations of enteropathy sensitive to CMP or in non-IgE-mediated allergies.
AAAAI and I-FPIES 2017^13^	*Not reported as recommendation.* In infants with CM-induced FPIES, introduction of SF under a physician's supervision and vice versa.SF as an alternative, especially in infants older than 6 months; a risk of potential co-reactivity between patients with soy-induced FPIES and those with CM-induced FPIES.
CNSFP 2018^9^	Not recommended as first-line treatment in infant <6 months (an increased risk of cross-reaction and unclear effect of phytoestrogen on hormonal balance).
SEGHPN, AEPAP, SEPEAP, and SEICAP 2019^15^	Recommendation against the use in infants aged less than 6 months.
GPIFN and MAP 2019^10^	May be used over 6 months of age if non-sensitized on IgE testing
NICE 2019^18^	- Recommendation against the use as a first line and not in infants less than 6 months of age or in those with suspected soy allergy - Recommendation for use in some children over 6 months of age without soy allergy. - Impact of isoflavones with a weak estrogenic action and with a theorized hormonal effect on the reproductive system: no consensus.
ISPGHAN 2020^12^	Infants more than 6 months of age with mild to moderate reaction: in case of financial constraints.

Other mammalian milk formula (ie, goat's) for CMA	
SEICAP 2015^16^	Formulas based on extensive soy and meat (pig collagen) hydrolysates can be used (limited data on clinical effectiveness and nutritional safety).
CNSFP 2018^9^	*Not reported as recommendation.* Other mammalian milk, such as goat's or ewe's milk-based formulas, only after individual testing.
SEGHPN, AEPAP, SEPEAP, and SEICAP 2019^15^	Formulas from other mammals (goat, sheep, buffalo, mare, camel, donkey) not recommended.
Other guidelines^2^^,^^8^^,^^10^, ^11^, ^12^, ^13^, ^14^^,^^17^^,^^18^	Not reported.

Cow's milk dietary substitutes for CMA	
Other mammalian milk	
EWPGAG 2010^8^	Not nutritionally adequate. *Goat's milk* commonly provokes clinical reactions in more than 90% of children with CMA, *donkey's milk* in about 15% and has a high cost.
WAO 2010^2^	*Not reported as a recommendation.*The option of another milk should be weighed individually against allergy, clinical, and nutritional considerations.Goat's, ewe's and buffalo's milks: not recommended (risk of severe reactions).Camel's milk: a substitute for children after 2 years.Equine milks: substitutes, in particular for children with delayed-onset CMA.
ESPGHAN 2012^11^	Goat's- and sheep's-milk protein: strictly avoided (high cross-reactivity with CMP).Other mammalian proteins not recommended.
BSACI 2014^17^	Other mammalian milk: not recommended.
SEICAP 2015^16^	The use of unmodified milk from other mammals (eg, sheep, goat, etc.): not advisable (risk of cross-reactivity with the CMP).Equine milk (mare, donkey): an alternative (the fat contents must be balanced to meet the nutritional requirements of children).
AAAAI and I-FPIES 2017^13^	*Not reported as recommendation.*Goat and sheep milk: not recommended in patients with CM-FPIES (based on high homology of the protein sequences in these animal milks).Milks from donkeys, camels, or both: might be tolerated in patients with CM-FPIES (usually well tolerated in those with IgE-mediated CMA).
CNSFP 2018^9^	*Not reported as recommendation.*Goat's or ewe's milks: only after individual testing (higher risk of reacting to other mammalian milk).
SEGHPN, AEPAP, SEPEAP, and SEICAP 2019^15^	Milks from other mammals (goat, sheep, buffalo, mare, camel, donkey): should not be used.
NICE 2019^18^	Other mammalian milk proteins (including unmodified cow, sheep, buffalo, horse, or goat's milk): not recommended (not adequately nutritious to provide the sole food source for infants, and risk of possible allergenic cross-reactivity with CM or formulas based on other mammalian milk proteins).
ISPGHAN 2020^12^	Unmodified mammalian milk (cow, buffalo, donkey, goat or camel): not recommended in infants with proven CMA.
Other guidelines^10^^,^^14^	Not reported.

Plant-based drinks	
ESPGHAN 2012^11^	Industrial juices made of soy, rice, almond, coconut, or chestnut, improperly called ‘‘milks’: not recommended (unsuitable to meet infant nutritional needs).
BSACI 2014^17^	Alternative ‘milks’: • not a main drink under 1 year of age (can be used for cooking); a nutritionally complete formula preferably to 2 years of age, • use under the guidance of a dietitian in children (risk of deficiency of energy, protein, Ca, riboflavin, vitamin A and D, and essential fatty acids), with regular monitoring of weight and growth, and in older children and adults (to ensure adequate Ca intake), • not in families with financial constraints, • need to ensure that specific ingredients are not allergenic, • rice milk: not recommended <4.5 years (natural inorganic arsenic content)
SEICAP 2015^16^	Unmodified soy, as well as non-adapted rice milks: contraindicated (not meet the necessary metabolic requirements).
CNSFP 2018^9^	*Not reported as recommendation.* Vegetable drinks: not nutritionally suited to the exclusive or partial feeding of infants; as complementary food in an otherwise well-balanced diet.
SEGHPN, AEPAP, SEPEAP, and SEICAP 2019^15^	Plant-based milks (soy, rice, oat, almond, tiger nut etc.): not recommended.
GPIFN and MAP 2019^10^	Children under 4.5 years: rice milk beverage not recommended; replacement milks only fortified with 120 mg calcium per serving.
NICE 2019^18^	Alternative 'milk' beverages (ie, almond, oat, coconut, or rice milks): not suitable for use as an infant's main drink under one year of age (poor nutritional value compared with cow's milk).Rice milk: not advised before the age of 4.5 years (natural inorganic arsenic content).Lactose-free formulas: not recommended in suspected or confirmed CMA (contain intact CMP).
Other guidelines^2^^,^^8^^,^^12^, ^13^, ^14^	Not reported.

Cow's milk re-challenge to test for acquired tolerance	
EWPGAG 2010^8^	- A child fed with CM formula with mild-moderate symptoms: if the oral food challenge is positive, the child elimination diet and re-challenged after 6 months (a shorter period for GORD) and in any case, after 9–12 months of age. - A child fed with CM formula with severe symptoms: the OFC for tolerance acquisition performed not before 6–12 months after the last reaction. Child elimination of CM until 12 months of age, but in those with enterocolitis syndrome, until 2–3 years of age. - A breasted child with moderate-severe symptoms: food challenge after 6–12 months of avoidance. If lack of symptoms after the reintroduction of CM in mother's diet, the introduction of excluded foods one by one in the diet.
WAO 2010^2^	*Not reported as recommendation.* Re-evaluation of all dietary interventions and avoidance strategies with patients and their families on a yearly basis, ideally through an OFC carried out under medical. Convincing symptoms after accidental ingestion equivalent to positive OFC and reschedule of the follow-up procedure accordingly.
Finnish guidelines 2012^14^	Not discussed in CMA section but with regard to food allergies in general. - A follow up of a child with food allergy by the basic health service. In case of a serious allergy for an important food (milk, grain), a follow up at the specialist-level health service. - In milk allergy, a trial with small amount milk made at home at the age of 18 months. - If CMA first appeared in the form of a serious allergy symptom, then milk provocation at specialist-level health care. Return of eliminated foods into the diet tried at 6-month intervals during the first 3 years and then at 12-month intervals. - Child 5-year visit (if not earlier): the examination of diet to ascertain whether based on an elimination–provocation trial and assess a need for consultation with a specialist.
BSACI 2014^17^	- Reassessment of individuals at 6–12 monthly intervals from 12 months of age to assess for suitability of reintroduction. - The challenge food in CMA: either baked or fresh milk. Baked milk for initial use (less allergenic, reactions less likely severe). - Home reintroduction using a ladder approach in children who have had only mild symptoms (only cutaneous symptoms) on noteworthy exposure (eg, a mouthful of fresh milk) and no reaction to milk in the past 6 months and in IgE-mediated disease, a significant reduction in sIgE/SPT weal diameter.
AAAAI and I-FPIES 2017^13^	Evaluation of patients with FPIES at regular intervals according to the patient's age and food allergen to determine whether she or he is still allergic. Recognition the age of development of tolerance varies by type of food trigger and country of origin. Development of tolerance in patients with CM-FPIES at an earlier age than tolerance in cereal grain- or other solid food induced FPIES.
CNSFP 2018^9^	A challenge under medical supervision to test the tolerance of baked milk in children from 1 year of age. The appropriateness and timing of its introduction assessed individually.*Not reported as recommendation.* Infants with proven CMA: a CM-free diet until 9–12 months of age and for at least 6 months before attempting to reintroduce it.
SEGHPN, AEPAP, SEPEAP, and SEICAP 2019^15^	Periods of treatment with a CMP-free diet: from 3 to 6 months in mild forms, to up to 12 months in the most severe cases. Unfavorable response to reintroduction of CMP: periodical re-evaluation of tolerance every 6 to 12 months.Mild cases: testing for tolerance at home under medical supervision.Child with a personal history of atopy, immediate reactions (onset within 2 h from ingestion), FPIES and all severe forms of allergy: a sIgE test and/or a SPT before reintroducing CMP. Based on the results of specific IgE or SPT: tolerance tested in a hospital setting.
GPIFN and MAP 2019^10^	Confirmed CMA: CM-free diet until 9–12 months of age and for at least 6 months – with a support of a dietitian. Then a planned reintroduction or supervised challenge using a ladder approach to determine tolerance acquisition.No current AD and no history at any time of immediate onset symptoms: no need to test IgE or SPT: reintroduction at home, using a milk ladder.Current AD: check serum sIgE or SPT. If negative: and still no history at any age of immediate onset symptoms - reintroduction at home using a milk ladder. If positive, refer to local pediatric allergy service.History of immediate onset symptoms at any time: sIgE or SPT. If negative, referral to local allergy service for re- challenge. If positive or test not available, refer to local pediatric allergy service.
NICE 2019^18^	Re-testing: arranged every 12–18 months depending on local pathways and protocols.Strict adherence to a CM-free diet for the mother/infant until the child is 9–12 months old and for at least 6 months. If symptoms do not improve over this time: (1) and CMA no longer suspected, the mother/infant resume normal feeding - referral to a pediatrician if symptom persist; (2) and CMA still suspected, referral to an allergology specialist and seeking specialist advice to avoid soy protein and egg.Child with non-IgE-mediated allergy: following a CM-free diet, a planned home reintroduction of cow's milk into the mother's or infant's diet. Tolerance to CMP e assessed using a 'milk ladder' and monitoring the symptoms (baked milk products reintroduced first (heating reduces allergenicity)).Signs of current atopic eczema or any history at any time of immediate-onset symptoms: home reintroduction contradicted and referral to an allergy specialist for allergy testing.Established tolerance: greater exposure of less processed milk gradually encouraged, ending in the reintroduction of fresh CM. Oral antihistamines available at home, in case of symptoms on reintroduction.Symptoms return on reintroduction of CM: a CM-free diet continued, and re-evaluation after a 6 to 12 months.Confirmed IgE-mediated CMA: follow-up arranged by the specialist allergy service (may include serial allergy testing and subsequent OFC).
ISPGHAN 2020^12^	OFC required before reintroduction of the allergen after therapeutic elimination period to confirm development of tolerance. Infant with IgE-mediated CMA: the elimination diet continued for at least one year and re-evaluation every 6 months subsequently.
Other guidelines^11^^,^^16^	Not reported.

Introduction of complementary feeding in infants with CMA	
EWPGAG 2010^8^	- Home-made meals a dietary option after 4 months of age. - Breastfed infants: weaned as recommended for healthy children, but with avoidance of CM until 9–12 months of age and for at least 6 months from the beginning of the diet.
Finnish guidelines 2012^14^	*Not discussed in CMA section but with regard to food allergies in general.* - Introduction of additional foods in all children on a child-by-child basis beginning at the age of 4–6 months while breastfeeding is continued. Recommendation for introduction of wheat and oats before 6 months. - At about 1 year of age, to consider the start of eating the same food as the rest of the family. Regular and varied meals, and eating meals together additionally beneficial. School children's snacks require attention; healthy alternatives favored over soft drinks, candy, and doughnuts.
SEICAP 2015^16^	- Recommendation against delay of the introduction of complementary feeding. - Recommendation against elimination of beef from the diet. - Tolerance of thoroughly cooked dairy products by some patients with CMA. - Possible tolerance of the yoghurt by patients sensitized only to CM whey proteins.
AAAAI and I-FPIES 2017^13^	- Possible increased risk of having FPIES to other foods (most commonly rice or oats) in infants with CM-induced FPIES. - Recommendation against delay in introducing complementary foods past 6 months of life. A practical ordering for introducing solids at home start with fruits and vegetables, followed by other foods, such as red meats and cereals. In case of tolerance to a variety of early food proteins, more liberal subsequent introduction. - In an infant with severe CM-induced FPIES, consideration of supervised introduction of solids. Possibility of excluding the risk of severe reactions to small amounts in case of supervised OFCs to a mixture of several solids, followed by gradual build up to regular age-appropriate serving size at home. - Recommendation for a provision of guidance to parents during the introduction of complementary foods and consultation with a dietitian. - It is commonly recommended to introduce a new food as a single ingredient and, in the case of high-risk foods, to wait at least 4 days before introducing another food to observe for the development of a reaction. Even single-food elimination can be associated with significant nutritional deficiency. - Recommendation for foods that enhance developmental skills in infants (of various tastes and textures) to prevent aversive feeding behaviors and delay in the development of food acceptance and feeding skills. - Recommendation against routine avoidance of products with precautionary allergen labelling in patients with FPIES.
CNSFP 2018^9^	- Regular advice of adequate replacement of dairy products, if introduced solid foods. - Diversification not restricted except in cases of other proven food allergies. - Need of dietary advice even when CMA is outgrown.
SEGHPN, AEPAP, SEPEAP, and SEICAP 2019^15^	- Complementary feeding in children with CMA: adherence to the guidelines applied to any other child under similar circumstances, save for the exclusion of CMP from the diet. - No need to elimination of beef and similar meats, always well cooked.
NICE 2019^18^	- Recommendations on how to advise caregivers on sources of information and support, and how to check and interpret food labels and recognize food allergens in ingredients lists of food products (includes lists of alternative terms for specific food allergen, and advice on precautionary allergen labeling, such as 'may contain' or 'not suitable for' statements) included in the guidelines. - A consideration for avoidance of the loose foods (for example bought from markets or open bakeries) and foods imported from outside the EU, due to risk of lacking food ingredient labeling.
ISPGHAN 2020^12^	*Not reported as recommendation.* - Introduction of supplementary foods one at a time in small quantities, preferably during the breastfeeding but not before the infant is at least 17 weeks of age to prevent other allergies. - No evidence to suggest any protective effect of delaying introduction of solid foods, or even potentially allergenic foods, beyond age 4–6 months.
Other guidelines^2^^,^^10^^,^^11^^,^^17^	Not reported.

Allergen immunotherapy (eg, oral, sublingual, epicutaneous, baked milk diet)	
WAO 2010^2^	Recommendation against administration of OIT with CM in patients with IgE-mediated CMA outside of clinical research
BSACI 2014^17^	Oral tolerance induction as a novel treatment option to the small but clinically significant proportion of affected individuals whose CMA persists.
SEICAP 2015^16^	- OIT in IgE-mediated CMA: a promising treatment to achieve desensitization in most cases, inducing immune modulating changes, and promoting tolerance. - Always used in a center with experience in the management of OIT and with the capacity to deal with the possible adverse reactions. - Long-term controlled trials are needed before general use of OIT in patients with CMA. - The risk/benefit ratio of OIT in early infancy must be considered (an experience of spontaneous resolution of their IgE-mediated CMA vs. a need of regular exposure to the allergen in order to maintain tolerance). - Before starting treatment based on OIT for milk and with the purpose of determining the clinical reactivity threshold, a consideration of careful controlled exposure test. - A need for further exploration of immunotherapy with food allergens, although especially in subcutaneous and oral immunotherapy association with significant adverse effects.
Other guidelines^8^, ^9^, ^10^, ^11^, ^12^, ^13^, ^14^, ^15^^,^^18^	Not reported.

Management of anaphylaxis and other emergencies (eg, acute FPIES)	
WAO 2010^2^	Dietary elimination of the trigger food or foods for the primary management of FPIES and education of caregivers and other care providers regarding avoidance strategies**.**
BSACI 2014^17^	If a history of anaphylaxis, prescription of intramuscular adrenaline for emergency use.
SEICAP 2015^16^	*Diagnosis of patient with anaphylaxis is mentioned, but not the management.* AAF is recommended in severe cases of anaphylaxis.
SEGHPN, AEPAP, SEPEAP, and SEICAP 2019^15^	- Acute FPIES treated as a medical emergency with possibility to provide aggressive fluid resuscitation. Individual management of acute FPIES according to severity and review treatment strategies with the caregivers of each patient. Consideration of ondansetron as an adjunctive management of emesis. - Dietary elimination of the trigger food or foods for the primary management of FPIES and education of caregivers and other care providers regarding avoidance strategies. Infants with suspected CM-induced FPIES generally advised to avoid all forms of these foods, including baked and processed foods, unless already included in the diet. Introduction of baked CM and egg under physician supervision.
GPIFN and MAP 2019^10^	If severe IgE-mediated CMA – anaphylaxis, emergency treatment and admission.
NICE 2019^18^	- Immediate ambulance transfer to Accident and Emergency, if systemic symptoms or suspected anaphylaxis with or without angioedema. - Referral to a specialist allergy clinic for allergy testing to confirm the diagnosis and guide management, the urgency depending on clinical judgement, if a history of one or more severe systemic reactions. Whilst awaiting specialist assessment, consider referral to a pediatric dietitian. - Written advice given to parents/carers on prompt recognition and management of acute symptoms following accidental or new exposures. - Oral antihistamines available at home, in case of a return of symptoms on reintroduction or any accidental exposure. - AAF recommended in management.
Other guidelines^8^^,^^9^^,^^11^, ^12^, ^13^, ^14^	Not reported.

Nutritional deficiencies in CMA	
EWPGAG 2010^8^	Diets must be nutritionally balanced. A supplementation with Ca must be evaluated.
WAO 2010^2^	*Not reported as recommendation.* A balanced calorie-protein ratio, amino-acid composition and an adequate Ca source required. The major risk of imbalanced diets are rickets (described vitamin D deficiency rickets) and poor growth.
ESPGHAN 2012^11^	- Children with CMA beyond the first 12 months of age need individualized nutritional advice. Dietetic assessment is required to: (1) assess the intake of nutrients, especially proteins, Ca, vitamin D, and vitamin A, and (2) check a need for therapeutic formula or supplements to support normal growth for age. - Supervision of the diet by a specialist dietician/pediatrician trained in pediatric nutrition strongly recommended. - Chronic iron-deficiency anemia may be the sole manifestation of CMA in infants and children. - Failure to thrive is nonspecific but can have severe consequences for a growing child.
BSACI 2014^17^	A risk of a deficient calcium intake. Assessment of Ca intake and advise of dietary or pharmaceutical supplementation where appropriate, by dietitian.
SEICAP 2015^16^	- A risk of lesser intake of nutrients than recommended may affect growth and development. - In older children: individualized dietetic controls are sometimes needed to ensure an adequate intake of proteins, calcium and vitamins A and D, with periodic monitoring to make sure that growth is normal for the age of the patient.
CNSFP 2018^9^	- An assessment of Ca and vitamin D intakes and counseling to reach RDA for these nutrients in all children with CMA. Counseling should include the importance and sources of Ca intake, and the expected objectives and timeline. - The assessment of bone metabolism (BMD and metabolic bone profile) advised only if suspected bone fragility (fracture(s); rickets; CMA associated with another chronic disease or multiple food allergies; the association of low Ca intake, low vitamin D intake, low energy intake, period of rapid growth, and persisting CMA such as during eosinophilic esophagitis).
SEGHPN, AEPAP, SEPEAP, and SEICAP 2019^15^	Growth (weight, length/height, and head circumference) assessed at regular intervals based on national standards. Main nutrients of concern: calcium, protein, fat, vitamin D
NICE 2019^18^	- A risk of inadequate nutritional intake, malabsorption, and faltering growth in children if food allergens that contribute essential nutrients are eliminated (ie, iron). - Consideration of referral to a pediatric dietitian if IgE-mediated, and if non-IgE-mediated CMA suspected. Referral to a pediatric dietitian in case of confirmed mild-to-moderate non-IgE-mediated allergy,
Other guidelines^10^^,^^12^, ^13^, ^14^	Not reported.

Probiotics for CMA	
WAO 2010^2^	*Not reported as a recommendation.* Some studies suggested a positive effect of probiotic interventions on atopic dermatitis, but meta-analyses did not confirm it. More RCTs need to be conducted to elucidate whether probiotics are useful for the treatment of AD**.**
Finnish guidelines 2012^14^	*Not discussed in CMA section but with regard to food allergies in general. Lactobacillus rhamnosus* GG inhibits and ameliorates atopic eczema to some extent. There is no consistent evidence for the usefulness of probiotic bacteria in airways allergies.
Other guidelines^8^, ^9^, ^10^, ^11^, ^12^, ^13^^,^^15^, ^16^, ^17^, ^18^	Not reported.

Prebiotics for CMA	
Not reported in any guidelines.^2^^,^^8^, ^9^, ^10^, ^11^, ^12^, ^13^, ^14^, ^15^, ^16^, ^17^, ^18^	

Synbiotics for CMA	
Not reported in any guidelines.^2^^,^^8^, ^9^, ^10^, ^11^, ^12^, ^13^, ^14^, ^15^, ^16^, ^16^, ^17^, ^18^	

Polyunsaturated fatty acids (PUFAs) for CMA	
WAO 2010^2^	*Not reported as recommendation.* The use of PUFAs to treat CMA could be attempted in well-defined cases, but there is a need for more and comprehensive (pre-clinical) data for widespread recommendation.
Other guidelines^8^, ^9^, ^10^, ^11^, ^12^, ^13^, ^14^, ^15^, ^16^, ^17^, ^18^	Not reported.

Other non-pharmacological treatment methods (ie, Chinese herbal medicine) for CMA	
WAO 2010^2^	*Not reported as recommendation.* Studies are in the preclinical stage to treat food allergy with a traditional Chinese herbal remedy.
Other guidelines^8^, ^9^, ^10^, ^11^, ^12^, ^13^, ^14^, ^15^, ^16^, ^17^, ^18^	Not reported.

AAAAI, American Academy of Allergy, Asthma and Immunology; AAF, amino acid formula; AD, atopic dermatitis; AEPAP, Spanish Association of Paediatric Primary Care; BMD, bone mineral density; BSACI, British Society for Allergy and Clinical Immunology; CM, cow's milk; CMA, cow's milk allergy; CMP, cow's milk protein; CNSFP, Committee of Nutrition of the French Society of Paediatrics; EHCF, extensively hydrolyzed casein formula; EHF, extensively hydrolyzed formula; EHWF, extensively hydrolyzed whey formula; ESPGHAN, European Society of Paediatric Gastroenterology; EWGPAG, the Emilia-Romagna Working Group for Paediatric Allergy and that for Paediatric Gastroenterology; FPIES, food protein-induced enterocolitis syndrome; GOR, gastro-esophagal reflux; GORD, gastro-esophagal reflux disease; GPIFN, General Practice Infant Feeding Network;; I-FPIES, International Food Protein-Induced Enterocolitis Syndrome (FPIES) Association; ISPGHAN, Indian Society of Pediatric Gastroenterology, Hepatology and Nutrition; MAP, Milk Allergy in Primary; NICE, National Institute for Health and Care Excellence; OFC, oral food challenge; OIT, oral immunotherapy; PUFA, polyunsaturated fatty acids; RCT, randomized controlled trial; RDA, recommended dietary allowance; SEGHPN, Spanish Society of Paediatric Gastroenterology, Hepatology, and Nutrition; SEICAP, Spanish Society of Pediatric Allergy, Asthma and Clinical Immunology; SEPEAP, Spanish Society of Extra-hospital Paediatrics and Primary Health Care; SF, soy formula; sIgE, specific immunoglobulin E; SPT, skin prick tests, WAO, World Allergy Organization

#### Diagnosis

Recommendations for clinical history and physical examination to establish suspicion of CMA were presented in 9 guidelines.^8^^,^^10^, ^11^, ^12^, ^13^^,^^15^, ^16^, ^17^, ^18^ Use of oral food challenge (OFC) and/or home reintroduction of baked-milk for diagnosis of CMA was recommended mostly in cases with suspicion of non-IgE-mediated CMA in four guidelines;^10^^,^^11^^,^^17^^,^^18^ in 3 guidelines, it was advised regardless of IgE-mechanism,^8^^,^^11^^,^^12^^,^^14^ and, in 4 guidelines,^2^^,^^13^^,^^15^^,^^16^ in only defined specific cases. Skin prick or specific serologic IgE tests were recommended only if IgE-mediated CMA was suspected according to 9 guidelines;^2^^,^^10^, ^11^, ^12^, ^13^^,^^15^, ^16^, ^17^, ^18^ however, in 1 set of guidelines, it was recommended regardless of type of CMA reaction.^8^

#### Maternal elimination of cow's milk during breastfeeding

A continuation of breastfeeding with a maternal cow's milk elimination diet was recommended in 8 guidelines.^8^^,^^10^, ^11^, ^12^^,^^14^, ^15^, ^16^^,^^18^ Six of the included guidelines^10^^,^^11^^,^^13^^,^^15^^,^^16^^,^^18^ recommended against a maternal elimination diet if the infant was asymptomatic on breastfeeding alone; in an additional one,^8^ it was recommended against elimination diet in case of mild symptoms. Supplementation of the maternal elimination diet with calcium was recommended in 7 guidelines,^2^^,^^10^, ^11^, ^12^^,^^15^^,^^16^^,^^18^ including 3,^10^^,^^15^^,^^18^ that also recommended supplementation of vitamin D.

#### Use of cow's milk formula

Extensively hydrolyzed formulas (EHFs) were recommended as a first-line treatment in formula-fed children with CMA in 5 guidelines;^2^^,^^11^^,^^13^^,^^16^^,^^18^ however, in 3 guidelines,^10^^,^^12^^,^^15^ EHFs were only recommended for infants with mild-to moderate CMA. Amino acid formula was recommended in the management of children with severe CMA symptoms in 6 guidelines,^8^^,^^10^^,^^12^^,^^15^^,^^17^^,^^18^ and/or in those with a high risk of anaphylaxis according to 4 guidelines,^2^^,^^11^^,^^16^^,^^18^ and/or in case of no response to or refusing EHF according to 5 guidelines.^11^^,^^12^^,^^15^, ^16^, ^17^

#### Use of plant-based formula

Rice-based formula was recommended as the treatment of choice in selected infants according to 3 guidelines,^8^^,^^11^^,^^16^ and, in 1 additional set of guidelines,^15^ hydrolyzed rice formula was recommended as an alternative if the infant refuses or does not respond to EHF. Use of soy formula was not recommended in infants below 6 months of age in 10 guidelines.^8^, ^9^, ^10^, ^11^, ^12^^,^^14^, ^15^, ^16^, ^17^, ^18^

#### Use of plant based beverages and mammalian milks

Use of other mammalian milks was not recommended in children with CMA according to 7 guidelines;^8^^,^^11^^,^^12^^,^^15^, ^16^, ^17^, ^18^ however, in 1 of these,^16^ an exception was made for equine milk with modified fat content, which could be used as an alternative. Five guidelines^11^^,^^15^, ^16^, ^17^, ^18^ recommended against use of soy plant-based beverage in infants with CMA. According to 3 guidelines,^10^^,^^17^^,^^18^ use of rice plant-based beverage is not advised in children under 4.5 years of age. Two guidelines,^11^^,^^15^ recommend against any plant-based beverages.

#### Acquisition of tolerance

Eight guidelines^8^, ^9^, ^10^^,^^12^^,^^13^^,^^15^^,^^17^^,^^18^ recommended periodic re-assessments of acquisition of tolerance with oral food challenges in children with CMA; however, the recommended period varied across the documents. According to 4 guidelines,^8^^,^^13^^,^^15^^,^^16^ complementary feeding should be introduced similarly as in healthy children. Five guidelines recommended supervision of the elimination diet by a dietitian (ie, assessment of one or more specific nutrients intake).^10^^,^^11^^,^^16^, ^17^, ^18^

#### Pre-, pro- and synbiotic and nutrient supplementations

There were no recommendations with regard to probiotics, prebiotics, synbiotics, polyunsaturated fatty acids, or other non-pharmacological methods (ie, Chinese herbal medicine) for management of CMA.

**Figure:**
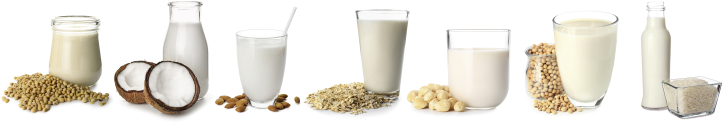

## Discussion

This systematic review assessed CMA guideline quality using the AGREE II instrument and summarized specific recommendations for the diagnosis and management of CMA. While the quality of the CMA guidelines published in the past 10 years varied, the median score for almost all domains exceeded 60%, except the rigor of development domain, that had the median score 30%; Q1-Q3: 15–67%. The clarity of presentation domain had the highest median score (92%; Q1-Q3: 81–100%). Three guidelines (BSACI, NICE, WAO) achieved the highest ratings (100%) in at least 3 domains and for the overall score.

### Agreement with other systematic reviews

Compared to the previous similar systematic review by Ruszczyński et al,^1^ which assessed CMA guidelines published from 2010 to November 2015, we included fewer full-text articles (12 compared to 15) despite the longer years of publication inclusion period. This is explained by our decision to only include the guidelines endorsed by the recognized scientific societies or organizations. Similar to Ruszczyński et al,^1^ we found the clarity of presentation to be the domain with the highest median score. We also found an improvement over time in the score for the applicability domain (68%, Q1-Q3: 57–75%) compared to the previous systematic review^1^ (32%, range: 6–100%).

In the recent, non-systematic review of CMA guidelines by Munblit et al,^19^ commercial involvement was reported as an important issue; 81% of authors in nine guidelines had financial conflict of interest with formula manufacturers and three CMA guidelines were directly supported by formula manufacturers. However, these 3 guidelines^20^, ^21^, ^22^ with financial support from pharmaceutical companies were not endorsed by any scientific organizations. In our review, the editorial independence (*domain 6*) was of good quality in the majority of included guidelines. Sixty-seven percent of authors in six guidelines^9^, ^10^, ^11^^,^^13^^,^^15^^,^^17^ declared conflicts of interest, in two^2^^,^^14^ individual conflict of interest was not reported, in the other four,^8^^,^^12^^,^^16^^,^^18^ there was nothing to declare.

The AGREE-II instrument is widely used to evaluate the methodological quality and transparency of guidelines that are used in clinical practice.^6^^,^^23^ It was also designed to inform development and reporting requirements for practice guidelines (ie, prioritization of the update of high quality guidelines and improvement of quality of guidelines during update, if necessary).^5^ Rigorous development and strategies for reporting are key predictors of successful implementation of the recommendations.^24^ Although the AGREE-II instrument focuses on methodological issues around guideline development and reporting, these issues are insufficient to ensure that recommendations are appropriate and valid, as methodological rigor and validity are not necessarily correlated.^25^ Therefore, when a clinician is choosing guidelines, some other factors may need to be considered according to the individual clinical situation, including guideline applicability (that may differ with regard to geographical region and/or available resources), scope of guidelines, and year of publication (preferably updated no later than 2 to 5 years from issue date).^5^

### Strengths and limitations

The search was limited to guidelines published in English only (*risk of language bias*). No blinding to the authors/organizations was implemented. However, the review team was well aware of available guidelines; thus, blinding, while feasible, might have been artificial. We used the AGREE II instrument to appraise all guidelines. However, the AGREE methodology has its limitations. For example, it does not provide a threshold for discrimination between good- and poor-quality guidelines, thus, the judgment is left to the appraisers. Of note, previous reviewers/appraisers, including members of the current panel,^1^ have provided input as to an appropriate quality threshold (ie, standardized domain score of above 60%).

Some of the authors who contributed to this systematic review were also authors of some of the included guidelines. However, the appraisal of methodological quality using the AGREE II instrument was performed by independent reviewers.

## Conclusions

The majority of the included CMA guidelines published from 2010 to 2020 were of good or very good quality. However, the weakest domain was the rigor of development, mostly due to the poorly described strengths and limitations of the body of evidence and the procedure for updating the guidelines.

## Abbreviations

AAAAI, Adverse Reactions to Food Committee of the American Academy of Allergy, Asthma and Immunology; AGREE, Appraisal of Guidelines for Research and Evaluation; AHRQ, Agency for Healthcare Research and Quality; BSACI, British Society for Allergy & Clinical Immunology; CMA, cow's milk allergy; CI, confidence interval; DRACMA, Diagnosis and Rationale for Action against Cow's Milk Allergy; EAACI, European Academy of Allergy and Clinical Immunology; EHFs, Extensively hydrolyzed formulas; ESPGHAN, European Society for Pediatric Gastroenterology, Hepatology, and Nutrition; FPIES, food protein-induced enterocolitis syndrome; GIM, Global Index Medicus; GIN, Guideline International Network; GPIFN, General Practice Infant Feeding Network, GRADE, Grading of Recommendations Assessment, Development and Evaluation; I-FPIES, International FPIES Association; ICC, intraclass correlation coefficient; ISPGHAN, Indian Society of Pediatric Gastroenterology; MAP, Milk Allergy in Primary; NICE, National Institute for Health and Care Excellence; OFC, oral food challenge; PRISMA, Preferred Reporting Items for Systematic Reviews and Meta-Analyses; SIGN, Scottish Intercollegiate Guidelines Network; TRIP, Turning Research into Practice; WAO, World Allergy Organization.

## Ethics approval

Ethics approval was not required for this study as it is a systematic review.

## Author’s contribution

HS, AS, AH, and MR initially conceptualized the study. AS, AH, MR, and LD contributed to data collection. AS, AH, and MR also performed data analysis and interpretation. AS and HS drafted the first version of the manuscript. All authors approved the final version of the manuscript.

## Funding

Medical University of Warsaw.

## Informed consent

Not applicable.

## Consent for publication

All authors approved the final version and its submission.

## Declaration of competing interest

AS has no potential conflict of interest to declare. AH has participated as a speaker for companies manufacturing infant formulas, ie, Danone, Nestle, Nestle Nutrition Institute, Nutricia, and Mead Johnson. MR has participated as a speaker for companies manufacturing infant formulas, ie, Danone, Nestle, Nestle Nutrition Institute, Nutricia, and Mead Johnson. LD have no conflicts of interest to declare. AF reports currently sponsored research by Danone/Nutricia, the Netherlands, Sanofi/Regeneron, U.S.A., Hipp, Germany, Ferrero, Italy. He is on advisory boards of Danone, Stallergenes, France, Menarini, Italy, Abbott, U.S.A., DBV, U.S.A. - France, Novartis, Switzerland, and Hipp. ANW has participated as consultant and/or speaker for companies manufacturing infant formulas, ie, Danone, Nestle, and Nestle Nutrition Institute. RS has participated as consultant and/or speaker for companies manufacturing infant formulas, ie, Abbott, Danone, Else Nutrition, Nestle, Nestle Nutrition Institute. JMS has participated as consultant for Abbott Nutrition and Nutricia. YV has participated as a clinical investigator, and/or advisory board member, and/or consultant, and/or speaker for Abbott Nutrition, Biogaia, By Heart, CHR Hansen, Danone, ELSE Nutrition, Friesland Campina, Nestle Health Science, Nestle Nutrition Institute, Nutricia, Mead Johnson Nutrition, Phathom Pharmaceuticals, United Pharmaceuticals (Novalac), Wyeth. CV has participated as a speaker for companies manufacturing infant formulas, ie, Danone, Nestle, Nestle Nutrition Institute, Nutricia, and Mead Johnson. CV also received grant funding from Reckitt and the National Peanut Board. HS has participated as consultant and/or speaker for companies manufacturing infant formulas, ie, Ausnutria, Cargill, Danone, Else Nutrition, Hipp, Nestle, Nestle Nutrition Institute. WAO DRACMA guideline group: S Arasi, S Bahna, Bognanni, J Brozek, D Chu, L Dahdah, E Galli, R Kamenwa, H Li, A Martelli, R Pawankar, H Schunemann, R Targino, L Terracciano, and A Warner have no conflicts to disclose. Relationships reported related to the submitted work: IJ Anstotegui – Abbott, Amgen, Astra Zeneca, Bayer, Bial, Faes Farma, Hikma, Menarini, Merck, Mundipharma, Roxall, Sanofi, Stallergenes, UCB. A Assa’ad – Aimmune Therapeutics, DBV Technologies, Astella, ABBVIE, Novartis, Sanofi, FARE, NIH and an intellectual property patent licensed to Hoth. R Berni Canani – Ch.Hansen, Danone, DVB, Humana, iHealth, Kraft Heinz, Mead Johnson, Nestlè, Novalac, Nutricia, Sanofi. M Bozzola – Danone C Dupont – Nestle Health Science, Nestle France, Nutricia, Novalac, Sodilac, Abbott, Danone, and stock ownership at DBV Technologies. M Ebisawa – DBV Technologies, Mylan, ARS Pharmaceuticals, Novartis. A Fiocchi – Abbott, Danone. G Lack – FARE, National Peanut Board (NPB), The Davis Foundation, Action Medical Research, UK Food Standards Agency, Medical Research Council, DBV Technologies, Mission Mighty Me, Novartis, Sanofi-Genyzme, Regeneron, ALK-Abello, Lurie Children’s Hospital. A Nowak-Wegrzyn – Nestle, Nutricia, Novartis, Gerber, Aimmune. N Papadopoulos – Novartis, Nutricia, HAL Allergy, Menarini/ Faes Farma, Sanofi, Mylan/Meda, Biomay, AstraZeneca, GSK, MSD, ASIT Biotech, Boehringer Ingelheim, Gerolymatos International SA, Capricare. M Said – Nestle, Nutricia, Abbott, Bayer for Anaphylaxis Australia. J Spergel – DBV Technologies, Regeneron, Sanofi, and Aimmune. H Szajewska – Ausnutria, Cargill, Danone, Else Nutrition, Hipp, Nestle, and Nestle Nutrition Institute. Y Vandenplas – Abbott Nutrition, Biogaia, Biocodex, By Heart, CHR Hansen, Danone, ELSE Nutrition, Friesland Campina, Hero, Hypocrata, Nestle Health Science, Nestle Nutrition Institute, Nutricia, Mead Johnson Nutrition, Orafti, Phacobel, Phathom Pharmaceuticals, Sari Husada, United Pharmaceuticals (Novalac), Wyeth, Yakult. C Venter – Reckitt Benckiser, Nestle Nutrition Institute, Danone, Abbott Nutrition, Else Nutrition, and Before Brands, DBV Technologies. S Waserman – Novartis-basic science work on peanut allergy, Aimmune-peanut OIT trial, Medical Advisor to Food Allergy Canada, and Pfizer, Bausch, Kaleo consultant for epinephrine autoinjectors. GWK Wong – Nestle, Danone.
