# Supplementary material for: World Allergy Organization (WAO) Diagnosis and Rationale for Action against Cow’s Milk Allergy (DRACMA) Guidelines update – IV – A quality appraisal with the AGREE II instrument
Source: World Allergy Organ J. 2022 Mar 2;15(2):100613. doi: 10.1016/j.waojou.2021.100613 (PMC9419447; doi:10.1016/j.waojou.2021.100613)
Supplement: Multimedia component 1 [file mmc1.docx]

**Supplementary Table 1. Characteristics of excluded papers with reasons**

| **Excluded article** | **Reason(s) for exclusion** |
| --- | --- |
| Abbott 2010^1^ | Not CMA guidelines: guidance of ELISA methods for the determination of quantitative ELISA allergens |
| Allen 2012^2^ | Not guidelines: clinical practice guide for GERD |
| Alonso-Lebrero 2018^3^ | Not CMA guidelines: only current opinions of experts |
| ASCIA 2016^4^ | Focused on anaphylaxis: lack of separate section on CMA |
| ASCIA 2016^5^ | Focused on skin prick tests manual for practitioners only |
| ASCIA 2017^6^ | Focused on unorthodox testing and allergic disorders treatment: lack of separate section on CMA |
| ASCIA 2016^7^, 2018^8^ | Focused on infant feeding and allergy prevention:  CMA prevention only |
| ASCIA 2018^9^ | Focused on eczema: lack of separate section on CMA |
| Baranov 2020^10^ | Focused on a single specific management (amino-acid formulas)  Language: Russian |
| Bartuzi 2017^11^ | Language: Polish  Food allergies guidelines: lack of separate section on CMA |
| Bingemann 2018^12^ | Not guidelines: a review  Focused on FPIES: lack of separate section on CMA |
| Bird 2020^13^ | Focused on conducting an oral food challenge: lack of separate section on CMA |
| Bocquet 2019^14^ | Focused on a single specific management |
| Boyce 2010^15^ | Focused on food allergy: lack of separate section on CMA |
| Boyce 2011^16^ | Not guidelines: summary of food allergy guidelines in the United States |
| Bręborowicz 2015^17^ | Language: Polish  Not guidelines: a review |
| Brooks 2017^18^ | Not CMA guidelines: a retrospective chart review of potential food-related allergic reactions |
| Bührer 2014^19^ | Language: German  Focused on nutrition recommendation for healthy children:  lack of separate section on CMA |
| Bunik 2011^20^ | Focused on allergic proctocolitis: lack of separate section on CMA |
| Campbell 2014^21^ | Not guidelines: a review |
| Campbell 2016^22^ | Abstract only  Focused on infant feeding and allergy prevention: lack of separate section on CMA |
| Campbell 2018^23^ | Not guidelines: a review  Focused on management of vomiting child |
| Chan 2013^24^ | Not CMA guidelines: focused on dietary exposures and allergy prevention |
| Chan 2016^25^ | Not CMA guidelines: focused on allergy prevention |
| Chang 2019^26^ | Focused on chronic cough and GERD in children: lack of separate section on CMA |
| Chantry 2015^27^ | Language: French  Not CMA guidelines: clinical guidelines on breastfeeding only |
| Comberiati 2015^28^ | Not CMA guidelines: a review  Focused on diagnosis and management of food allergy in children |
| Comité Nacional de Alergia^29^ | Language: Spanish |
| Critch 2011^30^ | Focused on infantile colic, not on CMA  *Separate section on hypoallergenic/hydrolyzed formula only* |
| Cubides-Munévar 2020^31^ | Language: Spanish |
| Darsow 2010^32^ | Focused on eczema: lack of separate section on CMA |
| D’Auria 2020^33^ | Not CMA guidelines: a review  Food allergy: lack of separate section on CMA |
| Davanzo 2015^34^ | Not CMA guidelines: position statement on breastfeeding |
| De Greef 2012^35^ | Not CMA guidelines: a review |
| Dempster 2011^36^ | An expert guide only, not endorsed by professional society  Focused on eczema: lack of separate section on CMA |
| Denis 2012^37^ | Language: French  Not guidelines: a review |
| Detzel 2012^38^ | Abstract only  Not guidelines: a systematic review  Focused on allergy prevention only |
| Di Mauro 2016^39^ | Not guidelines: consensus on prevention of food and airway allergy  Focused on prevention only  Lack of separate section on CMA |
| Doerfler 2015^40^ | Not CMA guidelines: a guide on dietary therapy in eosinophilic esophagitis |
| Doulgeraki 2017^41^ | Not guidelines: a practical guide only  Lack of separate section on CMA |
| Du Toit 2010^42^ | Not guidelines: a review |
| Dupont 2011^43^ | Language: French |
| Dupont 2017^44^ | Language: French  Not guidelines: a review |
| Dupont 2019^45^ | Review  Focused on FPIES and proctocolitis: lack of separate section on CMA |
| Dutau 2019^46^ | Language: French  Not guidelines: editorial/ a practical guide to the reintroduction of cow’s milk protein |
| Ebisawa 2017^47^ | Focused on food allergy: lack of separate section on CMA |
| Evans 2013^48^ | Not guidelines: a practical guide  Commentary on NICE guidelines |
| Feuille 2015^49^ | Not guidelines: a review  Focused on FPIES, allergic proctocolitis and enteropathies:  lack of separate section on CMA |
| Feuille 2016^50^ | Not guidelines: a review  Focused on oral immunotherapy for food allergies only |
| Fewtrell 2017^51^ | Not CMA guidelines: a position paper on complementary feeding, lack of separate section on CMA |
| Fiocchi 2015^52^ | Not guidelines: a review |
| Fiocchi 2016^53^ | Not guidelines: a review |
| Fiocchi 2016^54^ | Not guidelines: a review |
| Fleischer 2013^55^ | Not guidelines: a review on recommendations  Focused on primary prevention of allergic disease only |
| Fox 2011^56^ | Not guidelines: a review  Focused on food allergy: lack of separate section on CMA |
| Gamboni 2013^57^ | Not guidelines: a review |
| Garcia-Marcos 2018^58^ | Not guidelines: an editorial paper |
| Giovannini 2014^59^ | Not guidelines: a non-systematic review of nutritional aspects of food allergy |
| Gonsalves 2014^60^ | Not guidelines: a review  Focused on eosinophilic esophagitis: lack of separate section on CMA |
| Górriz Gil 2018^61^ | Not guidelines: a review/practical guide for otolaryngologists  Focused on Eeosinophilic esophagitis: lack of separate section on CMA |
| Groetch 2013^62^ | Not guidelines: a review  Focused on gastrointestinal allergy: lack of separate section on CMA |
| Groetsch 2021^63^ | Not guidelines: a narrative review |
| Guler 2020^64^ | Document not endorsed by a recognized scientific organization |
| Gutiérrez-Castrellón 2019^65^ | Language: Spanish  Recommendations on soy infant formula only |
| Halken 2021^66^ | Focused on prevention of food allergy only |
| Hilbig 2012^67^ | Language: German  Not CMA guidelines: focused on complementary feeding |
| Hirano 2020^68^ | Focused on eosinophilic esophagitis: lack of separate section on CMA |
| Hoyt 2015^69^ | Not guidelines: a review  Focused on a breastfeeding in prevention of food allergy only,  lack of separate section on CMA |
| Iskedjian 2011^70^ | Abstract only  Not guidelines: a systematic review  Focused on allergy prevention only |
| Jenzer 2012^71^ | Abstract only  Recommendations not endorsed by scientific organization  Focused on proton-pump inhibitors safety and nutrition support.  Lack of separate section on CMA |
| Johnson 2015^72^ | Not guidelines: a review/practical guide  Focused on infantile colic: lack of separate section on CMA |
| Jones 2016^73^ | Focused on management of *Helicobacter pylori*: lack of separate section on CMA |
| Joshi 2017^74^ | Not guidelines: a clinical practice guide |
| Joshi 2019^75^ | Not guidelines: guidelines summary  Focused on prevention of food allergy only |
| Kanabar 2017^76^ | Not guidelines: a practical guide/review  Focused on infant colic: lack of separate section on CMA |
| Kansu 2016^77^ | Document not endorsed by a recognized scientific organization. |
| Karásková 2017^78^ | Language: Czech  Not CMA guidelines: an overview of infant formulas available on Czech Republic market |
| Kipfer 2021^79^ | Focused on choice of formula in infants with CMA only |
| Kirkbright 2012^80^ | Not guidelines: a clinical practice guide  Anaphylaxis: lack of separate section on CMA |
| Kleine-Tebbe 2016^81^ | Language: German |
| Kolaĉek 2010^82^ | Language: Croatian  Not guidelines: a review |
| Kolaĉek 2011^83^ | Language: Croatian  Not CMA guidelines: recommendations on nutrition of healthy infants |
| Koletzko 2010^84^ | Language: German |
| Koletzko 2010^85^ | Language: German  Not CMA guidelines: recommendations on infant nutrition and nutrition for breastfeeding mothers |
| Koletzko 2013^86^ | Language: German  Not CMA guidelines:  update of recommendations on infant nutrition and nutrition for breastfeeding mothers |
| Koletzko 2014^87^ | Not CMA guidelines:  practice recommendation on long-chain polyunsaturated fatty acids in pregnancy, lactation, and infancy |
| Koletzko 2016^88^ | Language: German  Not CMA guidelines: recommendations on nutrition and physical activity of infants and breastfeeding women |
| Koplin 2018^89^ | Abstract only  Not guidelines: a randomized trial  Focused on peanut allergy only |
| Kopp 2015^90^ | Language: German  Focused on allergy prevention only |
| Krogulska 2015^91^ | Language: Polish  Not guidelines: a review  Focused on prevention of food allergy only |
| Laemmle-Ruff 2013^92^ | Not guidelines: clinical practice guide  Focused on anaphylaxis: lack of separate section on CMA |
| Lee 2010^93^ | Full version of guidelines is no longer available on the website. |
| Leonard 2012^94^ | Not guidelines: a review  Focused on FPIES: lack of separate section on CMA |
| Leonard 2015^95^ | Not guidelines: a review |
| Leonard 2016^96^ | Not guidelines: a review |
| Leonard 2018^97^ | Not CMA guidelines: a review of guidelines  Focused on FPIES: lack of separate section on CMA |
| Levin 2017^98^ | Not guidelines: an editorial  Focused on food allergy: lack of separate section on CMA |
| Li 2012^99^ | Language: Chinese |
| Li 2013^100^ | Language: Chinese |
| Lieberman 2010^101^ | Focused on anaphylaxis: lack of separate section on CMA |
| Lifschitz 2015^102^ | Not guidelines: a review |
| Lo Vecchio 2016^103^ | Focused on infant diarrhea: lack of separate section on CMA |
| Lopes Dos Santos 2016^104^ | Abstract only  Not guidelines: a practical guide? |
| Luyt 2016^105^ | Not guidelines: a cross-sectional study (a national audit) |
| Malaysian Health Technology Assessment Section 2018^106^ | Focused on atopic eczema: lack of separate section on CMA |
| Marangoni 2019^107^ | Not guidelines: a health professional’s guide |
| Martell 2014^108^ | Language: Spanish |
| Martorell 2017^109-112^ | Focused on oral immunotherapy only |
| Matricardi 2016^113^ | Not guidelines: user’s guide  Focused on molecular diagnostic only |
| Matthai 2011^114^ | Not guidelines: a review  Lack of separate section on CMA |
| Mazigh 2015^115^ | Language: French |
| Mehr 2019^116^ | Not guidelines: a review/guidelines summary and practice recommendations  Focused on FPIES: lack of separate section on CMA |
| Mendonça 2011^117^ | Language: Spanish  Not guidelines: a review  Focused on oral food challenges |
| Meyer 2012^118^ | Not guidelines: a review of current guidelines and a practical guide |
| Meyer 2018^119^ | Not guidelines: a review |
| Meyer 2020^120^ | Focused on non-IgE gastrointestinal allergies in breastfed infants: lack of separate section on CMA |
| Miceli Sopo 2019^121^ | Not guidelines: a review  Focused on FPIES: lack of separate section on CMA |
| Michelet 2017^122^ | Not guidelines: a review |
| Molina-Infante 2017^123^ | Not guidelines: a review |
| Montijo-Barrios 2014^124^ | Language: Spanish |
| Moya 2011^125^ | Abstract only  Not guidelines: a questionnaire survey/a cohort study? |
| Mukherjee 2019^126^ | Abstract only  Not guidelines: a proof of concept study |
| Munasir 2013^127^ | Not guidelines: a review |
| Muraro 2014^128^ | Not focused on CMA; only recommendations on cow’s milk substitutes |
| Netting 2017^129^ | Focused on prevention of food allergy only |
| Netting 2019^130^ | Not guidelines: a review  Focused on Food allergy:  lack of separate section on CMA |
| Netts 2017^131^ | Not guidelines: an editorial |
| NICE 2011^132^ | Focused on food allergy: excluded, because NICE CMA Guidelines (2019) included |
| NICE 2014^133^ | Focused on constipation: lack of separate section on CMA |
| NICE 2015^134^ | Focused on dyspepsia in adults: lack of separate section on CMA |
| NICE 2015, 2016^135,136^ | 2015 separate section, 2016 – lack of separate section |
| NICE 2017^137^ | Focused on infantile colic: lack of separate section on CMA |
| Nicolaou 2014^138^ | Not guidelines: a review |
| Nocerino 2015^139^ | Not guidelines: a review  Focused on Food allergy: lack of separate section on CMA |
| Nowak-Węgrzyn 2017^140^ | Not guidelines: a review only  Focused on FPIES: lack of separate section on CMA |
| Nowak-Węgrzyn 2019^141^ | Not guidelines: a review only |
| Nowak-Węgrzyn 2019^142^ | Not guidelines: a review only  Focused on immunotherapy only |
| Nowak-Węgrzyn 2020^143^ | Focused on FPIES: lack of separate section on CMA |
| O’Connor 2016^144^ | Consensus on female nutrition: lack of separate section on CMA |
| Pados 2020^145^ | Not guidelines: a review |
| Pajno 2018^146^ | Focused on immunotherapy only |
| Poussel 2016^147^ | Language: French  Focused on anaphylaxis: lack of separate section on CMA |
| Prell 2019^148^ | Language: German  Focused on prevention of allergy manifestations only |
| Rajani 2020^149^ | Not guidelines: a review only  Focused on food allergy: lack of separate section on CMA |
| Rancé 2011^150^ | Abstract only  Focused on food allergy: lack of separate section on CMA |
| Rancé 2011^151^ | Language: French  Focused on atopic dermatitis: lack of separate section on CMA |
| Recto 2017^152^ | Focused on prevention of allergic diseases only |
| Rosen 2018^153^ | Focus on gastroesophageal reflux, not CMA |
| Ruszczyński 2016^154^ | Not guidelines: a quality appraisal of 2010-2015 CMA guidelines |
| Sampson 2012^155^ | PRACTALL consensus report  lack of separate section on CMA |
| Sampson 2014^156^ | Focused on food allergy: lack of separate section on CMA |
| Sánchez 2014^157^ | Language: Spanish  Not guidelines: a review |
| Sekerel 2017^158^ | Expert panel only, not guidelines endorsed by scientific organization  Focused on Health economic burden on Turkish healthcare |
| Sicherer 2014^159^ | Not guidelines: a review |
| Sicherer 2017^160^ | Food allergy: lack of separate section on CMA |
| Sidbury 2014^161^ | Focused on atopic dermatitis: lack of separate section on CMA |
| Shaoul 2021^162^ | Language: Hebrew |
| SIGN 2011^163^ | Focused on atopic eczema: separate section: formula feeding in prevention only |
| Smith 2011^164^ | Not guidelines: a review article |
| Solé 2012^165^ | Language: Spanish |
| Song 2015^166^ | Language: Chinese  Prevention of food allergy only |
| Stagnaro-Green^167^ | Focused on thyroid disease management in pregnancy and postpartum: lack of separate section on CMA |
| Stear 2011^168^ | Not guidelines: a cross-sectional study |
| Subspecialty Group of, Gastroenterology  Society of Pediatrics, Chinese Medical Association  Subspecialty Group of, Pediatrics  Society of, Prenteral and  Enteral Nutrition, Chinese Medical Association 2012^169^ | Language: Chinese |
| Subspecialty Group of Immunology, The Society of Pediatrics, Chinese Medical Association; Subspecialty Group of Child Health Care, The Society of Pediatrics, Chinese Medical Association; Subspecialty Group of Digestion, The Society of Pediatrics, Chinese Medical Association; Editorial Board of Chinese Journal of Pediatrics^170^ | Language: Chinese |
| Szépfalusi 2015^171^ | Language: German |
| Tabbers 2014^172^ | Focused on functional constipation, not CMA |
| Taniuchi 2017^173^ | Not guidelines: a review  Focused on Immunotherapy only |
| Taylor 2014^174^ | An expert panel only  Establishment of reference doses for allergenic food residues only |
| Thyssen 2020^175^ | Focused on atopic dermatitis: lack of separate section on CMA |
| Turner 2018^176^ | Not guidelines: an editorial, referring to new BSACI guidance  Focused on prevention of food allergy only |
| Urisu 2011^177^ | Focused on food allergy: lack of separate section on CMA |
| Urisu 2014^178^ | Focused on food allergy: lack of separate section on CMA |
| Uscanga-Dominguez 2019^179^ | Focused on milk and its derivates in adults health and disease: lack of separate section on CMA |
| Vale 2015^180^ | Focused on prevention of anaphylaxis in schools, pre-schools and childcare: lack of separate section on CMA |
| Valovirta 2012^181^ | Language: Finnish  Abstract only  Focused on immunotherapy only |
| Van Neerven 2019^182^ | Not guidelines: an editorial |
| Vandenplas 2012^183^ | Not guidelines: a review |
| Vandenplas 2013^184^ | Not guidelines: the consensus-based algorithms |
| Vandenplas 2014^185^ | Not guidelines: a review  Focused on prevention of allergy only |
| Vandenplas 2014^186,187^ | Not official recommendations, developed to provide summary for a local purpose |
| Vandenplas 2015^188^ | Not guidelines: the consensus-based algorithms |
| Vandenplas 2015^189^ | Not guidelines: a review |
| Vandenplas 2015^190^ | Not guidelines: a review |
| Vandenplas 2015^191^ | Not guidelines: a workshop report only? |
| Vandenplas 2016^192^ | Not guidelines: a practical recommendation based on other consensus paper |
| Vandenplas 2016^193^ | Focused on functional gastrointestinal disorders, not CMA |
| Vandenplas 2016^194^ | Not endorsed by scientific organization |
| Vandenplas 2017^195^ | Not guidelines: a review |
| Vandenplas 2019^196^ | Not guidelines: a systematic review and expert consensus  Focused on prevention of allergy only |
| Vandenplas 2019^197^ | Not endorsed by scientific organization |
| Venter 2012^198^ | Not guidelines: an INDANA paper in diagnosis and management of food hypersensitivity  lack of separate section on CMA |
| Venter 2013^199^ | The most current version (Fox 2019) has been included. |
| Venter 2017^200^ | The most current version (Fox 2019) has been included. |
| Vlieg-Boerstra 2012^201^ | Abstract only |
| Vlieg-Boerstra 2013^202^ | Language: Dutch |
| Walsh 2014^203^ | Not guidelines: a practical algorithms/ an adjunct to the 2011 NICE guidelines |
| Walsh 2016^204^ | Not guidelines: a practical guide |
| Wang 2013^205^ | Language: Chinese |
| Wollenberg 2018^206^ | Focused on atopic eczema: lack of separate section on CMA |
| World Allergy Organization 2012^207^ | Language: Chinese  NO FULL TEXT |
| Yanagida 2018^208^ | Not guidelines: a letter to the editor, a cohort study  A three-level, stepwise, food challenge only |

BSACI, British Society for Allergy and Clinical Immunology; CMA, cow’s milk allergy; FPIES, Food Protein-Induced Enterocolitis Syndrome; GERD, gastroesophageal reflux disease; NICE, National Institute for Health and Care Excellence.

References:

1. Abbott M, Hayward S, Ross W, et al. Validation procedures for quantitative food allergen ELISA methods: Community guidance and best practices. *Journal of AOAC Int.* 2010;93(2):442-450.

2. Allen K, Ho S. Gastro-oesophageal reflux in children What’s the worry? *Aust Fam Physician.* 2012;41:268-272.

3. Alonso-Lebrero E, Bento L, Martorell-Aragonés A, Ribeiro L. Iberian consensus on cow's milk allergy: The CIBAL Study. *Allergol Immunopathol (Madr).* 2018;46(6):517-532.

4. Australian Society of Clinical Immunology and Allergy (ASCIA). Information for Health Professionals. Anaphylaxis Clinical Update. 2016.

5. Australian Society of Clinical Immunology and Allergy (ASCIA). Skin Prick Testing for the Diagnosis of Allergic Diseases. A manual for practitioners. 2016.

6. Australian Society of Clinical Immunology and Allergy (ASCIA). Unorthodox Testing and Treatment for Allergic Disorders. 2017.

7. Australian Society of Clinical Immunology and Allergy (ASCIA). Guidelines Infant Feeding and Allergy Prevention. 2016.

8. Australian Society of Clinical Immunology and Allergy (ASCIA). Information for Health Professionals. Infant Feeding and Allergy Prevention Clinical Update. 2018.

9. Australian Society of Clinical Immunology and Allergy (ASCIA). GUIDE Stepwise management of eczema (atopic dermatitis). 2018.

10. Baranov AA, Namazova-Baranova LS, Alexeeva AA, et al. Amino acid formulas in patients with food allergy. *Pediatricheskaya Farmakologiya.* 2020;17(6):536-546.

11. Bartuzi Z, Kaczmarski M, Czerwionka-Szaflarska M, et al. The diagnosis and management of food allergies. Position paper of the Food Allergy Section the Polish Society of Allergology. *Postepy Dermatol Alergol.* 2017;34(5):391-404.

12. Bingemann TA, Sood P, Järvinen KM. Food Protein-Induced Enterocolitis Syndrome. *Immunol Allergy Clin North Am.* 2018;38(1):141-152.

13. Bird JA, Leonard S, Groetch M, et al. Conducting an Oral Food Challenge: An Update to the 2009 Adverse Reactions to Foods Committee Work Group Report. *J Allergy Clin Immunol Pract.* 2020;8(1):75-90.

14. Bocquet A, Dupont C, Chouraqui JP, et al. Efficacy and safety of hydrolyzed rice-protein formulas for the treatment of cow's milk protein allergy. *Arch Pediatr.* 2019;26(4):238-246.

15. Boyce JA, Assa'ad AW, Jones SM. et al. Guidelines for the diagnosis and management of food allergy in the United States: Report of the NIAID-sponsored expert panel. *J Allergy Clin Immunol.* 2010;126 Suppl 6:S1-58.

16. Boyce JA, Assa'a A, Burks AW, et al. Guidelines for the diagnosis and management of food allergy in the United States: Summary of the NIAID-Sponsored Expert Panel Report. *Nutrition.* 2011;27(2):253-267.

17. Bręborowicz A, Sobkowiak P. Postępowanie diagnostyczne w alergii na białka mleka krowiego u dzieci. *Alergia Astma Immunologia.* 2015;20(1):17-23.

18. Brooks C, Coffman A, Erwin E, Mikhail I. Diagnosis and treatment of food allergic reactions in pediatric emergency settings. *Ann Allergy, Asthma Immunol.* 2017;119(5):467-468.

19. Bührer C, Genzel-Boroviczény O, Jochum F, et al. Nutrition of healthy infants. Recommendations of the Nutrition Committee of the German Pediatric Society. *Monatsschr Kinderheilkd.* 2014;162(6):527-538.

20. Bunik M, Chantry CJ, Howard CR, et al. ABM clinical protocol #24: Allergic proctocolitis in the exclusively breastfed infant. *Breastfeeding Med.* 2011;6(6):435-440.

21. Campbell D. Cow's milk allergy in infants: Diagnosis and management. *Prescriber.* 2014;25(9):11-14.

22. Campbell D, Vale S, Smith J, et al. ASCIA guidelines for infant feeding and allergy prevention. *Intern Med J.* 2016;46:6. https://doi.org/10.1111/imj.5-13197.

23. Campbell C, Slater Y. Approach to the vomiting child. *Paediatr Child Health (United Kingdom).* 2018;28(11):500-506.

24. Chan ES, Cummings C, Feldman M, et al. Dietary exposures and allergy prevention in high-risk infants. *Paediatr Child Health (Canada).* 2013;18(10):545-549.

25. Chan AWM, Chan JKC, Tam AYC, Leung TF, Lee TH. Guidelines for allergy prevention in Hong Kong. *Hong Kong Med J.* 2016;22(3):279-285.

26. Chang AB, Oppenheimer JJ, Kahrilas PJ, et al. Chronic Cough and Gastroesophageal Reflux in Children: CHEST Guideline and Expert Panel Report. *Chest.* 2019;156(1):131-140.

27. Chantry AA, Monier I, Marcellin L. Breastfeeding (part one): Frequency, benefits and drawbacks, optimal duration and factors influencing its initiation and prolongation. Clinical guidelines for practice. *J Gynecol Obstet Biol Reprod.* 2015;44(10):1071-1079.

28. Comberiati P, Cipriani F, Schwarz A, Posa D, Host C, Peroni DG. Diagnosis and treatment of pediatric food allergy: An update. *Ital J Pediatr.* 2015;41(1). https://doi.org/10.1186/s13052-014-0108-0.

29. Comité Nacional de Alergia. [Food allergy in children: recommendations for diagnosis and treatment]. *Arch Argent Pediatr.* 2018;116(1):s1-s9.

30. Critch J. Infantile colic: Is there a role for dietary interventions? *Paediatr Child Health.* 2011;16(1):47-49.

31. Cubides-Munevar AM, Linero-Terán AS, Saldarriaga-Vélez MA, et al. Alergia a la proteína de leche de vaca. Enfoque diagnóstico y terapéutico. *Rev Col Gastroenterol.* 2020;35(1):92-103.

32. Darsow U, Wollenberg A, Simon D, et al. ETFAD/EADV eczema task force 2009 position paper on diagnosis and treatment of atopic dermatitis. *J Eur Acad Dermatol Venereol.* 2010;24(3):317-328.

33. D'Auria E, Pendezza E, Zuccotti GV. Personalized Nutrition in Food Allergy: Tips for Clinical Practice. *Front Pediatr.* 2020;8. https://doi.org/10.3389/fped.2020.00113.

34. Davanzo R, Romagnoli C, Corsello G. Position Statement on Breastfeeding from the Italian Pediatric Societies. *Ital J Pediatr.* 2015;41(1). https://doi.org/10.1186/s13052-015-0191-x.

35. De Greef E, Hauser B, Devreker T, et al. Diagnosis and management of cow's milk protein allergy in infants. *World J Pediatr.* 2012;8(1):19-24.

36. Dempster J, Jani B, Daly T. Managing eczema in children - A treatment update. *J Fam Pract.* 2011;60(11):660-668.

37. Denis M, Loras-Duclaux I, Lachaux A. Cow's milk protein allergy through human milk. *Arch Pediatr.* 2012;19(3):305-312.

38. Detzel P, Spieldenner J, Heil-Ruess M, et al. Systematic review of the recommendations on the prevention of allergic manifestations in children. *World Allergy Organ J.* 2012;5:S160-S161.

39. Di Mauro G, Bernardini R, Barberi S, et al. Prevention of food and airway allergy: Consensus of the Italian Society of Preventive and Social Paediatrics, the Italian Society of Paediatric Allergy and Immunology, and Italian Society of Pediatrics. *World Allergy Organ J.* 2016;9(1). https://doi.org/10.1186/s40413-016-0111-6.

40. Doerfler B, Bryce P, Hirano I, Gonsalves N. Practical approach to implementing dietary therapy in adults with eosinophilic esophagitis: The Chicago experience. *Dis Esophagus.* 2015;28(1):42-58.

41. Doulgeraki AE, Manousakis EM, Papadopoulos NG. Bone health assessment of food allergic children on restrictive diets: a practical guide. *J Pediatr Endocrinol Metabol.* 2017;30(2):133-139.

42. du Toit G, Meyer R, Shah N, et al. Identifying and managing cow's milk protein allergy. *Arch Dis Child Educ Pract Ed.* 2010;95(5):134-144.

43. Dupont C, Chouraqui JP, de Boissieu D, et al. [Dietetic treatment of cow's milk protein allergy]. *Arch Pediatr.* 2011;18(1):79-94.

44. Dupont C, Soulaines P. Update on dietary management of cow's milk allergy. *Arch Pediatr.* 2017;24(12):1350-1357.

45. Dupont C. Food Protein-Induced Enterocolitis Syndrome and Proctocolitis. *Ann Nutr Metab.* 2019;73 Suppl 4:8-16.

46. Dutau G, Lavaud F. Practical guide to the reintroduction of cow's milk proteins. *Rev Fr Allergol.* 2019;59(1):1-2.

47. Ebisawa M, Ito K, Fujisawa T. Japanese guidelines for food allergy 2017. *Allergology Int.* 2017;66(2):248-264.

48. Evans S, Tuthill D. Practical guide to dietary management of cow's milk allergy. *Paediatr Child Health (United Kingdom).* 2013;23(8):367-369.

49. Feuille E, Nowak-Wegrzyn A. Food Protein-Induced Enterocolitis Syndrome, Allergic Proctocolitis, and Enteropathy. *Curr Allergy Asthma Rep.* 2015;15(8):50. https://doi.org/10.1007/s11882-015-0546-9.

50. Feuille E, Nowak-Wegrzyn A. Oral Immunotherapy for Food Allergies. *Ann Nutr Metab.* 2016;68 Suppl 1:19-31.

51. Fewtrell M, Bronsky J, Campoy C, et al. Complementary Feeding: A Position Paper by the European Society for Paediatric Gastroenterology, Hepatology, and Nutrition (ESPGHAN) Committee on Nutrition. *J Pediatr Gastroenterol Nutr.* 2017;64(1):119-132.

52. Fiocchi A, Dahdah L, Albarini M, Martelli A. Cow's milk allergy in children and adults. *Chem Immunol Allergy*. 2015;101:114-123.

53. Fiocchi A, Dahdah L, Bahna SL, et al. Doctor, when should I feed solid foods to my infant? *Curr Opin Allergy Clin Immunol.* 2016;16(4):404-411.

54. Fiocchi A, Dahda L, Dupont C, et al. Cow's milk allergy: towards an update of DRACMA guidelines. *World Allergy Organ J.* 2016;9(1):1-11.

55. Fleischer DM, Spergel JM, Assa'ad AH, Pongracic JA. Primary prevention of allergic disease through nutritional interventions. *J Allergy Clin Immunol Pract.* 2013;1(1):29-36.

56. Fox AT, Lloyd K, Arkwright PD, et al. The RCPCH care pathway for food allergy in children: An evidence and consensus based national approach. *Arch Dis Child.* 2011;96 Suppl 2:i25-i29.

57. Gamboni SE, Allen KJ, Nixon RL. Infant feeding and the development of food allergies and atopic eczema: An update. *Australas J Dermatol.* 2013;54(2):85-89.

58. Garcia-Marcos L. This is what scientific societies are for. The CIBAL consensus. *Allergol Immunopathol (Madr).* 2018;46(6):515-516.

59. Giovannini M, D'Auria E, Caffarelli C, et al. Nutritional management and follow up of infants and children with food allergy: Italian Society of Pediatric Nutrition/Italian Society of Pediatric Allergy and Immunology Task Force Position Statement. *Ital J Pediatr.* 2014;40. https://doi.org/10.1186/1824-7288-40-1.

60. Gonsalves N, Kagalwalla AF. Dietary treatment of eosinophilic esophagitis. *Gastroenterol Clin North Am.* 2014;43(2):375-383.

61. Gorriz Gil C, Matallana Royo V, Alvarez Montero O, et al. Eosinophilic esophagitis: an underdiagnosed cause of dysphagia and food impaction to be recognized by otolaryngologists. *HNO.* 2018;66(7):534-542.

62. Groetch M, Henry M, Feuling MB, Kim J. Guidance for the nutrition management of gastrointestinal allergy in pediatrics. *J Allergy Clin Immunol Pract.* 2013;1(4):323-331.

63. Groetch M, Baker MG, Durban R, et al. The practical dietary management of food protein-induced enterocolitis syndrome. *Ann Allergy Asthma Immunol.* 2021. https://doi.org/10.1016/j.anai.2021.03.007.

64. Guler N, Cokugras FC, Sapan N, et al. Diagnosis and management of cow's milk protein allergy in Turkey: Region-specific recommendations by an expert-panel. *Allergol Immunopathol (Madr).* 2020;48(2):202-210.

65. Gutiérrez-Castrellón P, Vázquez-Frías R, Jiménez-Gutiérrez C, et al. Recomendaciones sobre la utilización de las fórmulas infantiles con proteína aislada de soya en la alimentación del lactante. Documento de posición basado en la evidencia. *Gac Med Mex.* 2019;155:S1-S30.

66. Halken S, Muraro A, de Silva D, et al. EAACI guideline: Preventing the development of food allergy in infants and young children (2020 update). *Pediatr Allergy Immunol.* 2021. https://doi.org/10.1111/pai. 13496.

67. Hilbig A, Lentze MJ, Kersting M. Introduction and composition of complementary feeding. Scientific evidence and practical guidelines in Germany. *Monatsschr Kinderheilkd.* 2012;160(11):1089-1095.

68. Hirano I, Chan ES, Rank MA, et al. AGA institute and the joint task force on allergy-immunology practice parameters clinical guidelines for the management of eosinophilic esophagitis. *Ann Allergy Asthma Immunol.* 2020;124(5):416-423.

69. Hoyt AE, Medico T, Commins SP. Breast Milk and Food Allergy: Connections and Current Recommendations. *Pediatr Clin North Am.* 2015;62(6):1493-1507.

70. Iskedjian M, Berbari J, Navarro V, et al. Systematic review of the guidelines on the prevention of allergic manifestations in children. *Value Health.* 2011;14(7):A501.

71. Jenzer H, Sadeghi L, Krause C, et al. Adverse digestibility effects, drug-food interactions and long-term safety of proton pump inhibitors. *Eur J Hosp Pharm.* 2012;19(2):187-188.

72. Johnson JD, Cocker K, Chang E. Infantile Colic: Recognition and Treatment. *Am Fam Physician.* 2015;92(7):577-582.

73. Jones NL, Koletzko S, Goodman K, et al. Joint ESPGHAN/NASPGHAN Guidelines for the Management of Helicobacter pylori in Children and Adolescents (Update 2016). *J Pediatr Gastroenterol Nutr.* 2017;64(6):991-1003.

74. Joshi P, Frith K. Assessing and managing IgE-mediated food allergies in children. *Med Today.* 2017;18(3):37-43.

75. Joshi PA, Smith J, Vale S, Campbell DE. The Australasian Society of Clinical Immunology and Allergy infant feeding for allergy prevention guidelines. *Med J Aust.* 2019;210(2):89-93.

76. Kanabar D. Management of infantile colic in practice. *Prescriber.* 2017;28(10):13-18.

77. Kansu A, Yuce A, Dalgic B, et al. Consensus statement on diagnosis, treatment and follow-up of cow's milk protein allergy among infants and children in Turkey. *Turk J Pediatr.* 2016;58(1):1-11.

78. Karásková E. Infant formulas - Current recommendations. *Pediatrie pro Praxi.* 2017;18(1):186-189.

79. Kipfer S, Goldman RD. Formula choices in infants with cow's milk allergy. *Can Fam Physician.* 2021;67(3):180-182.

80. Kirkbright SJ, Brown SG. Anaphylaxis--recognition and management. *Aust Fam Physician.* 2012;41(6):366-370.

81. Kleine-Tebbe J, Waßmann-Otto A, Mönnikes H. [Food Allergy and Intolerance : Distinction, Definitions and Delimitation]. *Bundesgesundheitsblatt Gesundheitsforschung Gesundheitsschutz.* 2016;59(6):705-722.

82. Kolaček S, Barbarič I, Despot R, et al. Nutrition of healthy infants: Recommendations of the Croatian Society of Paediatric Gastroenterology, Hepatology and Nutrition. *Paediatr Croat.* 2010;54(1):53-56.

83. Kolacek S. [Food hypersensitivity in children]. *Acta Med Croatica.* 2011;65(2):155-161.

84. Koletzko S, Niggemann B, Friedrichs F, Koletzko B. Approach for suspected cow's milk protein allergy in infants. *Allergo Journal.* 2010;19(8):529-534.

85. Koletzko B, Brönstrup A, Cremer M, et al. Infant nutrition and nutrition for breastfeeding mothers : Recommendations - A consensus paper commissioned by the German"Young Families' Network" (Netzwerk Junge Familie). *Monatsschr Kinderheilkd.* 2010;158(7):679-689.

86. Koletzko B, Bauer CP, Brönstrup A, et al. Infant nutrition and nutrition for breastfeeding mothers: Updated practice recommendations of the network "healthy Start - Young Family Network" a project from in FORM. *Monatsschr Kinderheilkd.* 2013;161(3):237-246.

87. Koletzko B, Boey CCM, Campoy C, et al. Current information and asian perspectives on long-chain polyunsaturated fatty acids in pregnancy, lactation, and infancy: Systematic review and practice recommendations from an early nutrition academy workshop. *Ann Nutr Metab.* 2014;65(1):49-80.

88. Koletzko B, Bauer CP, Cierpka M, et al. Nutrition and physical activity of infants and breastfeeding women: Updated recommendations by “Healthy Start – Young Family Network” an initiative from IN FORM. *Monatsschr Kinderheilkd.* 2016;164(9):771-798.

89. Koplin J, Peters R, Gardiner K, Allen K, Curtis N. Infant peanut consumption, adherence to peanut introduction guidelines, and peanut allergy in infants born between 2013 and 2016. *Allergy: European Journal of Allergy and Clinical Immunology.* 2018;73:840. https://doi.org/ 10.1111/all.13540.

90. Kopp MV. New guidelines on allergy prevention - A pediatric viewpoint. *Allergologie.* 2015;38(8):385-390.

91. Krogulska A. Prevention of food allergy - Current recommendations and new opportunities. *Pediatr Pol.* 2015;90(6):451-458.

92. Laemmle-Ruff I, O’Hehir R, Ackland M, Tang M. Anaphylaxis Identification, management and prevention. *Aust Fam Physician.* 2013;42:38-42.

93. Lee BW, Aw MM, Chiang WC, et al. Academy of medicine, Singapore-Ministry of Health clinical practice guidelines: management of food allergy. *Singapore Med J.* 2010;51(7):599-607.

94. Leonard SA, Nowak-Wegrzyn A. Clinical diagnosis and management of food protein-induced enterocolitis syndrome. *Curr Opin Pediatr.* 2012;24(6):739-745.

95. Leonard SA, Caubet JC, Kim JS, et al. Baked milk- and egg-containing diet in the management of milk and egg allergy. *J Allergy Clin Immunol Pract.* 2015;3(1):13-23.

96. Leonard SA, Nowak-Wegrzyn AH. Baked Milk and Egg Diets for Milk and Egg Allergy Management. *Immunol Allergy Clin North Am.* 2016;36(1):147-159.

97. Leonard SA, Pecora V, Fiocchi AG, Nowak-Wegrzyn A. Food protein-induced enterocolitis syndrome: A review of the new guidelines. *World Allergy Organ J.* 2018;11(1). http://dx.doi.org/10.1186/s40413-017-0182-z.

98. Levin M, Goga A, Doherty T, et al. Allergy and infant feeding guidelines in the context of resource-constrained settings. *Journal of Allergy and Clinical Immunology.* 2017;139(2):455-458.

99. Li HQ. [Intensive reading of World Allergy Organization (WAO) Diagnosis and Rationale for Action against Cow's Milk Allergy (DRACMA) guideline]. *Zhonghua Er Ke Za Zhi.* 2012;50(7):516-518.

100. Li HQ. [Reading and interpreting evidence based recommendations for the diagnosis and management of cow's milk allergy in Chinese infants]. *Zhonghua Er Ke Za Zhi.* 2013;51(3):187-188.

101. Lieberman P, Nicklas RA, Oppenheimer J, et al. The diagnosis and management of anaphylaxis practice parameter: 2010 Update. *J Allergy Clin Immunol.* 2010;126(3):477-480.

102. Lifschitz C, Szajewska H. Cow's milk allergy: evidence-based diagnosis and management for the practitioner. *Eur J Pediatr.* 2015;174(2):141-150.

103. Lo Vecchio A, Vandenplas Y, Benninga M, et al. An international consensus report on a new algorithm for the management of infant diarrhoea. *Acta Paediatr.* 2016;105(8):e384-e389.

104. Lopes Dos Santos J. Update on Atopic Dermatitis-Perspective of the paediatrician. *Eur J Pediatr* 2016;175(11):1405-1406.

105. Luyt D, Krishnan MT, Huber P, Clark A. Practice of the Treatment of Milk Allergy in the UK: A National Audit. *Int Arch Allergy Immunol.* 2016;169(1):62-68.

106. Malaysian Health Technology Assessment Section (MaHTAS). Clinical Practice Guidelines. Management of Atopic Eczema. 2018.

107. Marangoni F, Pellegrino L, Verduci E, et al. Cow's Milk Consumption and Health: A Health Professional's Guide. *J Am Coll Nutr.* 2019;38(3):197-208.

108. Martell JAO, Hernandez REH. Diagnostic approach and management of cow's-milk protein allergy: Latin America guidelines, an International and multidisciplinary approach (GL-APLV). *Rev Investig Clin.* 2014;66:S5-S8.

109. Martorell A, Alonso E, Echeverría L, et al. Oral immunotherapy for food allergy: A Spanish guideline. Egg and milk immunotherapy Spanish guide (ITEMS GUIDE). Part 2: Maintenance phase of cow milk (CM) and egg oral immunotherapy (OIT), special treatment dosing schedules. Models of dosing schedules of OIT with CM and EGG. *Allergol Immunopath.* 2017;45(5):508-518.

110. Martorell A, Alonso E, Echeverría L, et al. Oral immunotherapy for food allergy: A Spanish guideline. Immunotherapy egg and milk Spanish guide (items guide). Part I: Cow milk and egg oral immunotherapy: Introduction, methodology, rationale, current state, indications contraindications and oral immunotherapy build-up phase. *Allergol Immunopath.* 2017;45(4):393-404.

111. Martorell A, Alonso E, Echeverría L, et al. Oral immunotherapy for food allergy: A spanish guideline. immunotherapy egg and milk spanish guide (ITEMS guide). part I: Cow milk and egg oral immunotherapy: Introduction, methodology, rationale, current state, indications, contraindications, and oral immunotherapy build-up phase. *J Investig Allergol Clin Immunol.* 2017;27(4):225-237.

112. Martorell A, Alonso E, Echeverría L, et al. Oral immunotherapy for food allergy: A spanish guideline. egg and milk immunotherapy Spanish guide (ITEMS GUIDE). part II: Maintenance phase of cow milk (CM) and egg oral immunotherapy (OIT), special treatment dosing schedules. models of dosing schedules of OIT with CM and egg. *J Investig Allergol Clin Immunol.* 2017;27(5):279-290.

113. Matricardi PM, Kleine-Tebbe J, Hoffmann HJ, et al. EAACI Molecular Allergology User's Guide. *Pediatr Allergy Immunol.* 2016;27 Suppl 23:1-250.

114. Matthai J. Chronic and persistent diarrhea in infants and young children: Status statement. *Indian Pediatrics.* 2011;48(1):37-42.

115. Mazigh S, Yahiaoui S, Ben Rabeh R, Fetni I, Sammoud A. [Diagnosis and management of cow's protein milk allergy in infant]. *Tunis Med.* 2015;93(4):205-211.

116. Mehr S, Campbell DE. Food protein-induced enterocolitis syndrome: guidelines summary and practice recommendations. *Med J Aust.* 2019;210(2):94-99.

117. Mendonça RB, Cocco RR, Sarni ROS, Solé D. Teste de provocação oral aberto na confirmação de alergia ao leite de vaca mediada por IgE: qual seu valor na prática clínica? *Rev Paul Pediatr.* 2011;29:415-422.

118. Meyer R, Venter C, Fox AT, Shah N. Practical dietary management of protein energy malnutrition in young children with cow's milk protein allergy. *Pediatr Allergy Immunol.* 2012;23(4):307-314.

119. Meyer R, Groetch M, Venter C. When Should Infants with Cow's Milk Protein Allergy Use an Amino Acid Formula? A Practical Guide. *J Allergy Clin Immunol Pract.* 2018;6(2):383-399.

120. Meyer R, Chebar Lozinsky A, Fleischer DM, et al. Diagnosis and management of Non-IgE gastrointestinal allergies in breastfed infants-An EAACI Position Paper. *Allergy.* 2020;75(1):14-32.

121. Miceli Sopo S, Gelsomino M, Rivetti S, Del Vescovo E. Food Protein-Induced Enterocolitis Syndrome: Proposals for New Definitions. *Medicina (Kaunas).* 2019;55(6). http://dx.doi.org/10.1111/pai.13489.

122. Michelet M, Schluckebier D, Petit LM, Caubet JC. Food protein-induced enterocolitis syndrome - a review of the literature with focus on clinical management. *J Asthma Allergy.* 2017;10:197-207.

123. Molina-Infante J, Gonzalez-Cordero PL, Arias A, Lucendo AJ. Update on dietary therapy for eosinophilic esophagitis in children and adults. *Expert Rev Gastroenterolol Hepatol.* 2017;11(2):115-123.

124. Montijo-Barrios E, Lopez-Ugalde MV, Ramirez-Mayans J, et al. Diagnostic approach and management of cow's-milk protein allergy: Latin America guidelines, an International and multidisciplinary approach. *Rev Investig Clin.* 2014;66:S9-S72.

125. Moya M, Dreborg S, R GH, et al. Results of an EPA advisory group discussion on the management of cow's milk allergy in infants by paediatricians in primary care. *Acta Paediatr.* 2011;100:22. http://dx.doi.org/10.1111/j.1651-2227.2011.02484.x.

126. Mukherjee S, Harding J, Daniel E, et al. Computer based diagnosis and management algorithm for cow's milk protein allergy (CMPA) in primary care in United Kingdom-a proof of concept study. *Allergy.* 2019;74:357. https://doi.org/ 10.1111/ all.13960.

127. Munasir Z, Muktiarti D. The management of food allergy in Indonesia. *Asia Pacific Allergy.* 2013;3(1):23-28.

128. Muraro A, Werfel T, Hoffmann-Sommergruber K, et al. EAACI Food Allergy and Anaphylaxis Guidelines: Diagnosis and management of food allergy. *Allergy.* 2014;69(8):1008-1025.

129. Netting MJ, Campbell DE, Koplin JJ, et al. An Australian Consensus on Infant Feeding Guidelines to Prevent Food Allergy: Outcomes From the Australian Infant Feeding Summit. *J Allergy Clin Immunol Pract.* 2017;5(6):1617-1624.

130. Netting MJ, Allen KJ. Reconciling breast-feeding and early food introduction guidelines in the prevention and management of food allergy. *J Allergy Clin Immunol.* 2019;144(2):397-400.

131. Netts P, Michaelis LJ. An interpretation of the new international MAP guideline for the management of Milk Allergy in Primary Care. *Clin Transl Allergy.* 2017;7(1).

132. The National Institute for Health and Care Excellence (NICE). Food allergy in under 19s: assessment and diagnosis. 23 February 2011.

133. The National Institute for Health and Care Excellence (NICE). Constipation in children and young people. 13 May 2014.

134. The National Institute for Health and Care Excellence (NICE). Dyspepsia and gastro-oesophageal reflux disease in adults. 23 July 2015.

135. The National Institute for Health and Care Excellence (NICE). Gastro-oesophageal reflflux disease in children and young people: diagnosis and management. 14 January 2015.

136. The National Institute for Health and Care Excellence (NICE). Gastro-oesophageal reflux in children and young people. 28 January 2016.

137. The National Institute for Health and Care Excellence (NICE). Colic - infantile. 2017.

138. Nicolaou N, Tsabouri S, Priftis KN. Reintroduction of cow's milk in milk-allergic children. *Endocr Metab Immune Disord Drug Targets.* 2014;14(1):54-62.

139. Nocerino R, Pezzella V, Cosenza L, et al. The controversial role of food allergy in infantile colic: evidence and clinical management. *Nutrients.* 2015;7(3):2015-2025.

140. Nowak-Wegrzyn A, Jarocka-Cyrta E, Moschione Castro A. Food Protein-Induced Enterocolitis Syndrome. *J Investig Allergol Clin Immunol.* 2017;27(1):1-18.

141. Feuille E, Nowak-Wegrzyn A. Medical Algorithms: Recognizing and treating food protein-induced enterocolitis syndrome. *Allergy.* 2019;74(10):2019-2022.

142. Nowak-Wegrzyn A, Sato S, Fiocchi A, Ebisawa M. Oral and sublingual immunotherapy for food allergy. *Curr Opin Allergy Clin Immunol.* 2019;19(6):606-613.

143. Nowak-Wegrzyn A, Cianferoni A, Bird JA, Fiocchi A, Caubet JC, Medical Advisory Board of the International FA. Managing food protein-induced enterocolitis syndrome during the coronavirus disease 2019 pandemic: Expert recommendations. *Ann Allergy Asthma Immunol.* 2020;125(1):14-16.

144. O'Connor DL, Blake J, Bell R, et al. Canadian Consensus on Female Nutrition: Adolescence, Reproduction, Menopause, and Beyond. *J Obstet Gynaecol Can.* 2016;38(6):508-554.

145. Pados BF, Davitt ES. Pathophysiology of Gastroesophageal Reflux Disease in Infants and Nonpharmacologic Strategies for Symptom Management. *Nurs Womens Health.* 2020;24(2):101-114.

146. Pajno GB, Fernandez-Rivas M, Arasi S, et al. EAACI Guidelines on allergen immunotherapy: IgE-mediated food allergy. *Allergy.* 2018;73(4):799-815.

147. Pouessel G, Deschildre A. [Anaphylaxis in children: What pediatricians should know]. *Arch Pediatr.* 2016;23(12):1307-1316.

148. Prell C, Bührer C, Jochum F, et al. Infant nutrition based on protein hydrolysates for risk reduction of allergic manifestations: Statement of the Nutrition Committee of the German Society for Pediatric and Adolescent Medicine (DGKJ). *Monatsschr Kinderheilkd..* 2019;167(3):246-250.

149. Rajani PS, Martin H, Groetch M, Järvinen KM. Presentation and Management of Food Allergy in Breastfed Infants and Risks of Maternal Elimination Diets. *J Allergy Clin Immunol Pract.* 2020;8(1):52-67.

150. Rancé F. Atopic dermatitis and food allergy: When and how to test. *Clin Transl Allergy.* 2011;1 (conference abstract).

151. Rancé F, Bidat E, Deschildre A. Clinical signs, diagnosis and management of allergy to cow milk proteins according to the international recommendations of DRACMA. *Rev Fr Allergol.* 2011;51(5):506-511.

152. Recto MST, Genuino MLG, Castor MAR, et al. Dietary primary prevention of allergic diseases in children: The Philippine guidelines. *Asia Pac Allergy.* 2017;7(2):102-114.

153. Rosen R, Vandenplas Y, Singendonk M, et al. Pediatric Gastroesophageal Reflux Clinical Practice Guidelines: Joint Recommendations of the North American Society for Pediatric Gastroenterology, Hepatology, and Nutrition and the European Society for Pediatric Gastroenterology, Hepatology, and Nutrition. *J Pediatr Gastroenterol Nutr.* 2018;66(3):516-554.

154. Ruszczyński M, Horvath A, Dziechciarz P, Szajewska H. Cow's milk allergy guidelines: a quality appraisal with the AGREE II instrument. *Clin Exp Allergy.* 2016;46(9):1236-1241.

155. Sampson HA, Gerth van Wijk R, Bindslev-Jensen C, et al. Standardizing double-blind, placebo-controlled oral food challenges: American Academy of Allergy, Asthma & Immunology-European Academy of Allergy and Clinical Immunology PRACTALL consensus report. *J Allergy Clin Immunol.* 2012;130(6):1260-1274.

156. Sampson HA, Aceves S, Bock SA, et al. Food allergy: a practice parameter update-2014. *J Allergy Clin Immunol.* 2014;134(5):1016-1025.

157. Sanchez J, Restrepo MN, Mopan J, Chinchilla C, Cardona R. [Milk and egg allergy: diagnosis, management and implications for Latin America]. *Biomedica.* 2014;34(1):143-156.

158. Sekerel BE, Seyhun O. Expert panel on practice patterns in the management of cow's milk protein allergy and associated economic burden of disease on health service in Turkey. *J Med Econ.* 2017;20(9):923-930.

159. Sicherer SH, Sampson HA. Food allergy: Epidemiology, pathogenesis, diagnosis, and treatment. *J Allergy Clin Immunol.* 2014;133(2):291-307.

160. Sicherer SH, Allen K, Lack G, et al. Critical Issues in Food Allergy: A National Academies Consensus Report. *Pediatrics.* 2017;140(2).

161. Sidbury R, Tom WL, Bergman JN, et al. Guidelines of care for the management of atopic dermatitis: Section 4. Prevention of disease flares and use of adjunctive therapies and approaches. *J Am Acad Dermatol.* 2014;71(6):1218-1233.

162. Shaoul R, Pevzner M, Goldshtein Z. [ALLERGY PREVENTION AND TREATMENT DURING THE FIRST YEAR]. *Harefuah.* 2021;160(3):161-166.

163. Scottish Intercollegiate Guidelines Network (SIGN). Management of atopic eczema in primary care. A national clinical guideline. March 2011.

164. Smith JD, Clinard V, Barnes CL. Pharmacists' guide to infant formulas for term infants. *J Am Pharm Assoc.* 2011;51(3):e28-e37.

165. Solé D, Amancio O.M.S., Jacob CMA, Cocco RR, Sarni R.O.S. Guia prático de diagnóstico e tratamento da Alergia às Proteínas do Leite de Vaca mediada pela imunoglobulina E. *Rev bras alerg imunopatol.* 2012;35(6):203-233.

166. Song T, Ahn K-M, Lee S-Y. Prevention of food allergy in infants: recommendation for infant feeding and complementary food introduction. *Allergy Asthma Respir Dis.* 2015;3(5):320-325.

167. Stagnaro-Green A, Abalovich M, Alexander E, et al. Guidelines of the American Thyroid Association for the diagnosis and management of thyroid disease during pregnancy and postpartum. *Thyroid.* 2011;21(10):1081-1125.

168. Stear G, Potter P, Labadarios D, Motala C. Management of food allergies in children in South Africa-determining aspects of the knowledge and practices of dietitians and medical practitioners. *Curr Allergy Clin Immunol.* 2011;24(3):145-155.

169. Subspecialty Group of G, Society of Pediatrics CMA, Subspecialty Group of P, Society of P, Enteral Nutrition CMA. [Infants with acute diarrhea during the clinical nutrition interventions path]. *Zhonghua er ke za zhi.* 2012;50(9):682-683.

170. Subspecialty Group of Immunology, The Society of Pediatrics, Chinese Medical Association; Subspecialty Group of Child Health Care, The Society of Pediatrics, Chinese Medical Association; Subspecialty Group of Digestion, The Society of Pediatrics, Chinese Medical Association; Editorial Board of Chinese Journal of Pediatrics. [Evidence based recommendations for the diagnosis and management of cow's milk allergy in Chinese infants]. *Zhonghua Er Ke Za Zhi.* 2013;51(3):183-186.

171. Szépfalusi Z, Spiesz K, Huttegger I. [Diagnostics and management of food allergies in childhood and adolescence]. *Wien Med Wochenschr.* 2015;165(17-18):354-360.

172. Tabbers MM, DiLorenzo C, Berger MY, et al. Evaluation and treatment of functional constipation in infants and children: evidence-based recommendations from ESPGHAN and NASPGHAN. *J Pediatr Gastroenterol Nutr.* 2014;58(2):258-274.

173. Taniuchi S, Takahashi M, Soejima K, Hatano Y, Minami H. Immunotherapy for cow's milk allergy. *Hum Vaccines Immunother.* 2017;13(10):2443-2451.

174. Taylor SL, Baumert JL, Kruizinga AG, et al. Establishment of Reference Doses for residues of allergenic foods: report of the VITAL Expert Panel. *Food Chem Toxicol.* 2014;63:9-17.

175. Thyssen JP, Berents T, Bradley M, et al. Clinical management of atopic dermatitis in adults: Mapping of expert opinion in 4 Nordic countries using a modified Delphi process. *Acta Derm. Vener.* 2020;100(1). https://doi.org/ 10.2340/00015555-3369.

176. Turner PJ, Feeney M, Meyer R, Perkin MR, Fox AT. Implementing primary prevention of food allergy in infants: New BSACI guidance published. *Clin Exp Allergy.* 2018;48(8):912-915.

177. Urisu A, Ebisawa M, Mukoyama T, Morikawa A, Kondo N. Japanese guideline for food allergy. *Allergol Int.* 2011;60(2):221-236.

178. Urisu A, Ebisawa M, Ito K, et al. Japanese Guideline for Food Allergy 2014. *Allergol Int.* 2014;63(3):399-419.

179. Uscanga-Dominguez LF, Orozco-Garcia IJ, Vazquez-Frias R, et al. Technical position on milk and its derivatives in adult health and disease from the Asociacion Mexicana de Gastroenterologia and the Asociacion Mexicana de Gerontologia y Geriatria. *Rev Gastroenterol Mex.* 2019;84(3):357-371.

180. Vale S, Smith J, Said M, Mullins RJ, Loh R. ASCIA guidelines for prevention of anaphylaxis in schools, pre-schools and childcare: 2015 update. *J Paediatr Child Health.* 2015;51(10):949-954.

181. Valovirta E, Korhonen K, Kuitunen M, et al. [Update on current care guidelines: Allergen specific immunotherapy]. *Duodecim.* 2012;128(1):108-109.

182. van Neerven RJJ, Savelkoul HFJ. The Two Faces of Cow's Milk and Allergy: Induction of Cow's Milk Allergy vs. Prevention of Asthma. *Nutrients.* 2019;11(8). https://doi.org/ 10.3390/nu11081945.

183. Vandenplas Y, Gottrand F, Veereman-Wauters G, et al. Gastrointestinal manifestations of cow's milk protein allergy and gastrointestinal motility. *Acta Paediatr.* 2012;101(11):1105-1109.

184. Vandenplas Y, Gutierrez-Castrellon P, Velasco-Benitez C, et al. Practical algorithms for managing common gastrointestinal symptoms in infants. *Nutrition.* 2013;29(1):184-194.

185. Vandenplas Y, Bhatia J, Shamir R, et al. Hydrolyzed formulas for allergy prevention. *J Pediatr Gastroenterol Nutr.* 2014;58(5):549-552.

186. Vandenplas Y, Abuabat A, Al-Hammadi S, et al. Middle East Consensus Statement on the Prevention, Diagnosis, and Management of Cow's Milk Protein Allergy. *Pediatr Gastroenterol Hepatol Nutr.* 2014;17(2):61-73.

187. Vandenplas Y, Abuabat A, Al-Hammadi S, et al. Correction: Middle East Consensus Statement on the Prevention, Diagnosis, and Management of Cow's Milk Protein Allergy. *Pediatr Gastroenterol Hepatol Nutr.* 2014;17(3):201-201.

188. Vandenplas Y, Alarcon P, Alliet P, et al. Algorithms for managing infant constipation, colic, regurgitation and cow's milk allergy in formula-fed infants. *Acta Paediatr.* 2015;104(5):449-457.

189. Vandenplas Y, Hauser B. An updated review on gastro-esophageal reflux in pediatrics. *Expert Rev Gastroenterol Hepatol.* 2015;9(12):1511-1521.

190. Vandenplas Y, Marchand J, Meyns L. Symptoms, Diagnosis, and Treatment of Cow's Milk Allergy. *Curr Pediatr Rev.* 2015;11(4):293-297.

191. Vandenplas Y, Dupont C, Eigenmann P, et al. A workshop report on the development of the Cow's Milk-related Symptom Score awareness tool for young children. *Acta Paediatr.* 2015;104(4):334-339.

192. Vandenplas Y. Algorithms for Common Gastrointestinal Disorders. *J Pediatr Gastroenterol Nutr.* 2016;63 Suppl 1:S38-40.

193. Vandenplas Y, Benninga M, Broekaert I, et al. Functional gastro-intestinal disorder algorithms focus on early recognition, parental reassurance and nutritional strategies. *Acta Paediatr.* 2016;105(3):244-252.

194. Vandenplas Y, Alturaiki MA, Al-Qabandi W, et al. Middle East Consensus Statement on the Diagnosis and Management of Functional Gastrointestinal Disorders in <12 Months Old Infants. *Pediatr Gastroenterol Hepatol Nutr.* 2016;19(3):153-161.

195. Vandenplas Y. Prevention and Management of Cow's Milk Allergy in Non-Exclusively Breastfed Infants. *Nutrients.* 2017;9(7):731. https://doi.org/ 10.3390/nu9070731.

196. Vandenplas Y, Latiff AHA, Fleischer DM, et al. Partially hydrolyzed formula in non-exclusively breastfed infants: A systematic review and expert consensus. *Nutrition.* 2019;57:268-274.

197. Vandenplas Y, Al-Hussaini B, Al-Mannaei K, et al. Prevention of Allergic Sensitization and Treatment of Cow's Milk Protein Allergy in Early Life: The Middle-East Step-Down Consensus. *Nutrients.* 2019;11(7):1444. https://doi.org/ 10.3390/nu11071444.

198. Venter C, Laitinen K, Vlieg-Boerstra B. Nutritional aspects in diagnosis and management of food hypersensitivity-the dietitians role. *J Allergy (Cairo).* 2012. https://doi.org/10.1155/2012/269376.

199. Venter C, Brown T, Shah N, Walsh J, Fox AT. Diagnosis and management of non-IgE-mediated cow's milk allergy in infancy - a UK primary care practical guide. *Clin Transl Allergy.* 2013;3(1):23.

200. Venter C, Brown T, Meyer R, et al. Better recognition, diagnosis and management of non-IgE-mediated cow's milk allergy in infancy: iMAP-an international interpretation of the MAP (Milk Allergy in Primary Care) guideline. *Clin Transl Allergy.* 2017;7:26.

201. Vlieg-Boerstra BJ, Wensing C, Meijer Y, et al. Development of a national guideline for the diagnosis of cow's milk allergy (CMA) in the Netherlands. *J Allergy Clin Immunol.* 2012;129(2):AB23.

202. Vlieg-Boerstra BJ, Wensing CL, Kneepkens CM, et al. [One guideline for diagnosing cow milk allergy; for primary, secondary and tertiary care in the Netherlands]. *Ned Tijdschr Geneeskd.* 2013;157(38):A6311.

203. Walsh J, Venter C, Brown T, Shah N, Fox AT. A practical approach for UK primary care on the management of cow's milk allergy in infants. *Br J Gen Pract.* 2014;64(618):48-49.

204. Walsh J, Meyer R, Shah N, Quekett J, Fox AT. Differentiating milk allergy (IgE and non-IgE mediated) from lactose intolerance: understanding the underlying mechanisms and presentations. *Br J Gen Pract.* 2016;66(649):e609-611.

205. Wang B, Zhan SY. [Issues to address in developing evidence based clinical practice guidelines: experience from evidence-based recommendations for the diagnosis and treatment of cow's milk protein allergy in infants]. *Zhonghua Er Ke Za Zhi.* 2013;51(3):165-167.

206. Wollenberg A, Barbarot S, Bieber T, et al. Consensus-based European guidelines for treatment of atopic eczema (atopic dermatitis) in adults and children: part I. *J Eur Acad Dermatol Venereol.* 2018;32(5):657-682.

207. World Allergy O. [The outline of World Allergy Organization (WAO) Diagnosis and Rationale for Action against Cow's Milk Allergy (DRACMA) guideline]. *Zhonghua er ke za zhi.* 2012;50(7):510-515.

208. Yanagida N, Minoura T, Kitaoka S, Ebisawa M. A three-level stepwise oral food challenge for egg, milk, and wheat allergy. *J Allergy Clin Immunol Pract.* 2018;6(2):658-660.
