# Supplementary material for: World Allergy Organization (WAO) Diagnosis and Rationale for Action against Cow’s Milk Allergy (DRACMA) Guidelines update – IV – A quality appraisal with the AGREE II instrument
Source: World Allergy Organ J. 2022 Mar 2;15(2):100613. doi: 10.1016/j.waojou.2021.100613 (PMC9419447; doi:10.1016/j.waojou.2021.100613)
Supplement: Multimedia component 2 [file mmc2.docx]

**Supplemental appendix 1. Search strategy**

1. MEDLINE via PubMed

| **Search** | **Query** |
| --- | --- |
| [#1](https://www.ncbi.nlm.nih.gov/pubmed) | milk OR cow* |
| [#2](https://www.ncbi.nlm.nih.gov/pubmed) | allerg* OR hypersensitivit* OR intolerance |
| [#3](https://www.ncbi.nlm.nih.gov/pubmed) | guideline* OR recommendation* OR (practice AND guidelines) OR consensus* OR (position AND paper) OR report OR (work AND group AND report) |
| [#4](https://www.ncbi.nlm.nih.gov/pubmed) | “last 10 years”[PDat] |
| #5 | Search #1 and #2 and #3 and #4 |

EMBASE

('milk'/exp OR 'milk' OR 'cow'/exp OR 'cow') AND (allerg* OR 'allergic reaction'/exp OR 'allergic reaction' OR 'intolerance'/exp OR 'intolerance' OR 'hypersensitivity'/exp OR 'hypersensitivity') AND ('practice guidelines'/exp OR 'practice guidelines' OR 'recommendations'/exp OR 'recommendations') AND (2010:py OR 2011:py OR 2012:py OR 2013:py OR 2014:py OR 2015:py OR 2016:py OR 2017:py OR 2018:py OR 2019:py OR 2020:py OR 2021:py)
